# Novel Stilbene-Nitroxyl Hybrid Compounds Display Discrete Modulation of Amyloid Beta Toxicity and Structure

**DOI:** 10.3389/fchem.2022.896386

**Published:** 2022-05-26

**Authors:** Silvia Hilt, Ruiwu Liu, Izumi Maezawa, Tatu Rojalin, Hnin H. Aung, Madhu Budamagunta, Ryan Slez, Qizhi Gong, Randy P. Carney, John C. Voss

**Affiliations:** ^1^ Department of Biochemistry and Molecular Medicine, University of California, Davis, Davis, CA, United States; ^2^ M.I.N.D. Institute and Department of Pathology and Laboratory Medicine, University of California, Davis, Davis, CA, United States; ^3^ Department of Biomedical Engineering, University of California, Davis, Davis, CA, United States; ^4^ Division of Cardiovascular Medicine, Department of Internal Medicine, School of Medicine, University of California, Davis, Davis, CA, United States; ^5^ Research Division, California Air Resource Board, Sacramento, CA, United States; ^6^ Department of Cell Biology and Human Anatomy, School of Medicine, University of California, Davis, Davis, CA, United States; ^7^ Paramag Biosciences Inc., Davis, CA, United States

**Keywords:** protein misfolding, protein aggregation, oxidative stress, EPR (electron paramagnetic resonance), circular dichroism (CD), amyloid beta peptide (Aβ), Alzheimer’s disease

## Abstract

Several neurodegenerative diseases are driven by misfolded proteins that assemble into soluble aggregates. These “toxic oligomers” have been associated with a plethora of cellular dysfunction and dysregulation, however the structural features underlying their toxicity are poorly understood. A major impediment to answering this question relates to the heterogeneous nature of the oligomers, both in terms of structural disorder and oligomer size. This not only complicates elucidating the molecular etiology of these disorders, but also the druggability of these targets as well. We have synthesized a class of bifunctional stilbenes to modulate both the conformational toxicity within amyloid beta oligomers (AβO) and the oxidative stress elicited by AβO. Using a neuronal culture model, we demonstrate this bifunctional approach has the potential to counter the molecular pathogenesis of Alzheimer’s disease in a powerful, synergistic manner. Examination of AβO structure by various biophysical tools shows that each stilbene candidate uniquely alters AβO conformation and toxicity, providing insight towards the future development of structural correctors for AβO. Correlations of AβO structural modulation and bioactivity displayed by each provides insights for future testing *in vivo*. The multi-target activity of these hybrid molecules represents a highly advantageous feature for disease modification in Alzheimer’s, which displays a complex, multifactorial etiology. Importantly, these novel small molecules intervene with intraneuronal AβO, a necessary feature to counter the cycle of dysregulation, oxidative stress and inflammation triggered during the earliest stages of disease progression.

## 1 Introduction

The amyloid beta peptide (Aβ) plays central role in the etiology of Alzheimer’s Disease (AD), representing the earliest and most validated marker for the disease (Selkoe and Hardy, 2016; Sperling et al., 2020). Despite decades of study, a poor understanding of the conformational/thermodynamic states of the soluble oligomeric Aβ peptide (AβΟ) continues to confound efforts to design interventions targeting this species. In recent years, clinical trials have focused on lowering extracellular Aβ by immunotherapy (Usman et al., 2021). The results from most studies have missed their defined endpoints, raising the question as to whether lowering extracellular Aβ alone is sufficient to alter disease progression. In addition, complications associated with targeting extracellular Aβ deposits immunotherapy are common, such as inadvertently generating AβO from deposits [e.g., “the dust raising effect” (Liu et al., 2012)].

AβOs are distinguished from the Aβ monomers or fibrillar deposits by their neurotoxicity (Gong et al., 2003; Haass and Selkoe, 2007; Guerrero-Muñoz et al., 2014) and association to pathology in AD brains and animal models (Kayed et al., 2003; Selkoe and Hardy, 2016). For example, cell culture studies blocking Aβ oligomerization show protection from toxicity while compounds that promote fibril formation can be protective (Necula et al., 2007; Bieschke et al., 2011; Dutta et al., 2017). Other studies have established that AβOs trigger a variety of downstream effects resulting in cognitive deficits and neuronal destruction (Shankar et al., 2008; Almeida et al., 2009; Cottart et al., 2010; Rege et al., 2014; Currais et al., 2016; Selkoe and Hardy, 2016). This includes measurements in animal and cell culture models demonstrating that AβO drives Tau phosphorylation and aggregation (Brouillette et al., 2012; Bloom, 2014; Dunning et al., 2016; Park et al., 2018).

The self-association of Aβ occurs through a complex interplay of different oligomeric sizes and peptide conformations, whose dynamic equilibrium is sensitive to the peptide concentration, length, modification and local environment (Cline et al., 2018). For example, smaller species have been identified as causing synaptic dysfunction, whereas larger oligomers are associated with overall neurotoxicity (Figueiredo et al., 2013). Thus, identifying a single pathogenic species *via* a defined size and conformational state common to the various intra- and extra-cellular milieus is unlikely. While it is not possible for synthetic AβO preps to fully recapitulate the heterogeneity and post-translational modifications of Aβ assemblies found *in vivo*, a multitude of studies suggest that preparations of mid-sized oligomers (>50 kDa) correlate strongest along various pathways of neurotoxicity and cellular dysregulation (Cline et al., 2018; Shea et al., 2019). These oligomers are structurally disordered, react with the conformational antibody A11, ranging in size from the dodecamer up to 150 kDa. The pathogenic conformation of the peptides within these assemblies is still unknown, although pathogenic conformations have been proposed including the β-sheet edge (Yoshiike et al., 2008), exposure of hydrophobic patches (Ladiwala et al., 2012), alpha-sheet secondary structure (Shea et al., 2019), or alternative states of the hairpin region of the peptide that correlate to a “toxic” turn (Murakami, 2014; Silverman et al., 2018).

AβOs in brain tissue are found in both extracellular and intracellular compartments (Oakley et al., 2006; Oddo et al., 2006; Pensalfini et al., 2014). While disease diagnosis relies on extracellular amyloid, the pool of intracellular Aβ may play a greater role in the initiation of AD (Takahashi et al., 2017). Intraneuronal Aβ precedes both intracellular NFT and extracellular amyloid deposits (Knobloch et al., 2007; Pensalfini et al., 2014) and has profound effects on neuronal health (Takahashi et al., 2017). There is also accumulating evidence that suggests that intraneuronal Aβ42 is a major risk factor for neuronal loss and a trigger for the Aβ cascade of pathological events (Kienlen-Campard et al., 2002). Because intraneuronal Aβ precedes extracellular amyloid deposits and pTau filament formation, small molecules with access to the brain and cell interior may have a superior potential to intervene at the earliest stages of AD. Small molecules also offer flexibility in the ability to engage more than one type of misfolded protein, which may be key as increasing evidence indicates a direct influence among the various misfolded proteins (Aβ, Tau, α-Syn, TDP-43) associated with neurodegenerative proteinopathies (Colom-Cadena et al., 2013; Limanaqi et al., 2020).

The multifactorial nature of AD is commonly recognized, implying the involvement of several neurobiological targets in the initiation and development of this neurodegenerative disease. This challenge may in part be related to the complexity and dynamic nature of the AβO, leading to a multitude of reported mechanisms for its cytotoxicity (Kayed and Lasagna-Reeves, 2012). One important mechanism involves the elevation of reactive oxygen species (ROS), as oxidative damage can be considered the earliest known biochemical marker in the AD brain (Gong et al., 2003). AβO triggers oxidative stress in neurons, whereas oxidative stress increases Aβ production and/or clearance (Jo et al., 2010; Sultana and Butterfield, 2010). This process propagates a circular cascade of increased Aβ and ROS production (Swerdlow et al., 2014), which can also trigger microglial activation and Tau phosphorylation and misfolding (Maccioni et al., 2010; Rudenko et al., 2019).

Stilbenes such as resveratrol have shown therapeutic potential in models for AD and other neurodegenerative diseases (Rege et al., 2014; Ahmed et al., 2017; Drygalski et al., 2018). For example, resveratrol promotes Aβ clearance (Marambaud et al., 2005; Moussa et al., 2017), alters Aβ structure and aggregation properties (Ladiwala et al., 2010; Ge et al., 2012; Fu et al., 2014), and attenuates inflammation and oxidative stress in models for dementia and neurodegenerative disease (Sarubbo et al., 2017; Wang et al., 2018). Support for clinical evaluation of resveratrol in AD treatment has been shown in Alzheimer’s mouse models (Karuppagounder et al., 2009; Capiralla et al., 2012; Ma et al., 2014). We have developed a new series of small molecules that alter both the conformational toxicity of AβO and provide potent, long-lived antioxidant activity. The molecules are constructed to contain a stilbene scaffold for AβO engagement and a nitroxide spin label for antioxidant activity. We have designated these bifunctional molecules as paramagnetic amyloid ligands (PALs). Here we show five chemically distinct PAL molecules for their effect on AβO structure and assembly, as well as their ability to protect against AβO toxicity and oxidative stress.

## 2 Materials and Methods

### 2.1 Materials

Amyloid beta (Aβ) peptide (1–40) was purchased from EZBiolab Inc., Carmel, IN, United States. Aβ^TOAC26^ was synthesized by Prof. Lorigan and co-workers at Miami University (Petrlova et al., 2012). Hoechst Blue 3342 nuclear stain and oligomer A11 polyclonal antibody were purchased from Thermo Fisher, Waltham, MA, United States. CellROX (Deep Red; λex/λem 640/665 nm) was purchased from Life Technologies, Carlsbad, CA, United States. Opti-Minimal Essential Medium (OPTIMEM) was purchased from Invitrogen/Life Technologies, United States. PBS pH 7.4 (-Calcium Chloride, -Magnesium Chloride), Opti-MEM^®^ I Reduced Serum Medium (no phenol red), DMEM (Dulbecco’s modified Eagle’s medium +4.5 g/L Glucose, L-Glutamine and 110 mg/L Sodium Pyruvate) and Fetal Bovine Serum (FBS) were purchased from Gibco (Carlsbad, CA, United States). 35 mm Glass Bottom Dishes (No. 1.5) purchased from MatTek. Trypan Blue Solution (0.4%) was purchased from Sigma-Aldrich. Resveratrol 3,4′-diacetate, 3-hydroxymethyl-(1-oxy-2,2,5,5 -tetramethylpyrroline, 3-(2′-Iodoacetamido)-2,2,5,5-tetramethyl-1-pyrrolidinyl-1-oxyl, (1-oxyl-2,2,5,5,-tetramethyl-Δ3-pyrroline)formaldehyde, 2,2,5,5- tetramethyl-3-pyrrolin-1-oxyl-3-carboxylic acid, free radical, were purchased from Toronto Research Chemicals, Toronto, Canada. Acetonitrile was purchased from Thermo Fisher Scientific (Houston, TX, United States). All other chemical reagents and solvents were purchased from Aldrich (Milwaukee, WI, United States).

### 2.2 Synthesis of Spin-Labeled Paramagnetic Amyloid Ligands

#### 2.2.1 Synthesis of PMT-301

The synthetic scheme of PMT-301 is shown in Supplementary Figure S1. In brief, resveratrol 3,4′-diacetate (8.3 mg, 0.0266 mmol), 3-hydroxymethyl-(1-oxy-2,2,5,5 -tetramethylpyrroline) (5 mg, 0.0294 mmol), triphenylphosphine (PPh_3_, 8.0 mg, 0.0305 mmol), and anhydrous tetrahydrofuran (THF, 0.2 ml) were combined into a 1.5 ml microcentrifuge tube. The reaction tube was then lowered into a 42-kHz sonication bath (Cole-Parmer) and sonicated for 2 min. While sonicating, diisopropyl azodicarboxylate (DIAD, 6.6 μl, 0.0335 mmol) was added to the reaction mixture. The reaction mixture was sonicated for 25 min. The reaction mixture was then added to a KOH solution (20 μl, 10% aqueous solution) and stirred for 30 min at room temperature. The solution was neutralized with 0.05% trifluroacetic acid (TFA) in acetonitrile and then submitted for purification with preparative reversed-phase high performance liquid chromatography (HPLC) using a C18 column (Vydac, 10 μm, 2.2 cm i.d. × 25 cm) and gradient of 25%–100% B over 34 min at a flow rate of 5 ml/min (solvent A, H_2_O/0.05% TFA; B, acetonitrile/0.05% TFA). The eluant was collected and lyophilized to give powder PMT-301. The chemical identity was confirmed with Orbitrap ESI-MS, with a calculated mass for C_23_H_26_NO_4_ of 380.19, and observed mass of 381.20 [M+1] and 382.20 [M+2].

#### 2.2.2 Synthesis of PMT-302

The synthetic scheme of PMT-302 is shown in Supplementary Figure S2. Cs_2_CO_3_ (20 mg, 0.0624 mmol) was added to a solution of resveratrol 3,4′-diacetate (13 mg, 0.0416 mmol) in anhydrous dimethylformamide (DMF, 1 ml) and the reaction mixture was stirred at room temperature for 45 min, 3-(2′-Iodoacetamido)-2,2,5,5-tetramethyl-1-pyrrolidinyl-1-oxyl (13.5 mg, 0.0415 mmol) was then added to the solution and the resulting mixture was stirred at room temperature overnight. The reaction mixture was added to KOH solution (20 μl, 10% aqueous solution) and then stirred for 30 min at room temperature. The solution was neutralized with acetic acid and then purified by HPLC using the above-mentioned conditions. The eluent was lyophilized to give powder PMT-302. The chemical identity was confirmed with Orbitrap ESI-MS, with a calculated mass for C_24_H_29_N_2_O_5_ of 425.21, and observed mass of 426.22 [M+1] and 427.22 [M+2].

#### 2.2.3 Synthesis of PMT-303

The synthetic scheme of PMT-303 is shown in Supplementary Figure S3. 2,2,5,5-Tetramethyl-3-pyrrolin-1-oxyl-3-carboxylic acid anhydride free radical was first prepared by adding *N,N′*-dicyclohexylcarbodiimide (DCC, 56 mg, 0.271 mmol) to a solution of 2,2,5,5- tetramethyl-3-pyrrolin-1-oxyl-3-carboxylic acid, free radical (100 mg, 0.542 mmol) in anhydrous dichloromethane (3 ml). The mixture was stirred at room temperature for 1 h. The precipitate was filtered out and the clear solution was concentrated and dried over vacuum to give the SL-anhydride. To a solution of resveratrol (25.3 mg, 0.11 mmol) in 1 ml anhydrous dimethyl sulfoxide (DMSO) in a round-bottomed flask, sodium hydride (11 mg, 60% dispersion in mineral oil) was added. The resulting mixture was stirred at room temperature for 20 min, followed by the addition of SL-anhydride (38.5 mg, 0.11 mmol). The reaction solution was stirred at room temperature for 2 h. The reaction was quenched with water (100 μl), then 5 ml of cold water (with 0.1% acetic acid) was added to the solution. The solid was separated by centrifuge, redissolved in 80% acetonitrile in water (with 0.05% TFA) and purified by HPLC as described above. The chemical identity was confirmed with Orbitrap ESI-MS, with a calculated mass for C_23_H_24_NO_5_ of 394.16, and observed mass of 395.18 [M+1] and 396.18 [M+2].

#### 2.2.4 Synthesis of PMT-401

The synthetic scheme of PMT-401 is shown in Supplementary Figure S4. Stannous chloride (4.74 g, 25 mmol) was added to a solution of (*E*)-4-(4-nitrostyryl) phenol (1.2 g, 5 mmol) in ethanol (40 ml) followed by the addition of concentrated hydrochloric acid (2.0 ml). The solution was refluxed for 3 h and then cooled down to room temperature stirring overnight. Dark brown precipitate was collected by filtration and washed with small amount of ethanol to give (*E*)-4-(4-aminostyryl) phenol as HCl salt, light brown powder, 850 mg, yield 68.4%. Orbitrap ESI-MS for C_14_H_13_NO 211.10, Found 212.10 [M+1]. To a mixture of (*E*)-4-(4-aminostyryl) phenol HCl salt (495.4 mg, 2.0 mmol), paraformaldehyde (600 mg, 20 mmol) and sodium cyanoborohydride (378 mg, 6.0 mmol), acetic acid (20 ml) was added. The resulting mixture was heated until the solution became clear and stirred at room temperature overnight. 200 ml of water was added to the reaction solution. Sodium carbonate was added to adjust the pH to 8–9. After extraction with dichloromethane (3 ml × 40 ml), the combined dichloromethane layer was washed with water and brine, and dried over anhydrous Na_2_SO_4_. The liquid was collected by filtration and concentrated *via* rotovap to give (*E*)-4-(4-(dimethylamino)styryl)phenol as a light grey solid, 144 mg, yield 30%. Orbitrap ESI-MS for C_16_H_17_NO 239.13, Found 240.14 [M+1]. To an 1.5 ml eppendorf tube was added (*E*)-4-(4-(dimethylamino)styryl)phenol (7.7 mg, 0.032 mmol), 3-hydroxymethyl-(1-oxy-2,2,5,5 -tetramethylpyrroline) (6.0 mg, 0.0352 mmol), triphenylphosphine (PPh_3_, 9.7 mg, 0.0368 mmol), and anhydrous tetrahydrofuran (THF, 0.3 ml). The reaction tube was then lowered into a 42-kHz sonication bath (Cole-Parmer) and sonicated for 2 min. While sonicating, diisopropyl azodicarboxylate (DIAD, 7.9 µl, 0.04 mmol) was added to the reaction mixture. The reaction mixture was sonicated for 15 min, repeated three times, total 45 min. The reaction mixture was diluted with 2 ml of 50% acetonitrile/water (0.05% TFA) and then submitted for HPLC purification as described above. The eluent was collected and lyophilized to yield PMT-401 as dark brown powder. Orbitrap ESI-MS for C_25_H_31_N_2_O_2_ 391.24, found 392.25 [M+1].

#### 2.2.5 Synthesis of PMT-402

The synthetic scheme of PMT-402 is shown in Supplementary Figure S5. PMT-402 was synthesized from (*E*)-4-(4-aminostyryl) phenol. Its HCl salt (200 mg) was suspended in ethanol (25 ml) and then K_2_CO_3_ aqueous solution was added until pH 9. Water (25 ml) was added to the suspension and mixed. After centrifuge, the solid was collected by filtration and washed with water, 70% ethanol in water and dried over vacuum to give (*E*)-4-(4-aminostyryl)phenol as brownish solid. A suspension of (*E*)-4-(4-aminostyryl)phenol (7.6 mg, 0.0357 mmol), (1-oxyl-2,2,5,5,-tetramethyl-Δ3-pyrroline)f ormaldehyde (6 mg, 0.0357 mmol) and p-toluenesulfonic acid (1 mg, 0.0058 mmol) in mixture of ethanol (1.5 ml) and anhydrous THF (0.8 ml) was sonicated for 5 min until the solution became clear and stirred at room temperature for additional 15 min. After the resulting solution was cooled down with ice-water bath, NaBH_4_ (27 mg, 0.714 mmol) was added. After the mixture was stirred at room temperature overnight, water (20 ml) was added, followed by addition of acetic acid to adjust the pH to 6. The precipitate was collected after centrifuge and redissolved in 80% acetonitrile in water (with 0.05% TFA) for HPLC purification as described above. The eluent was collected and lyophilized to yield PMT-402 as brownish powder. The chemical identity was confirmed with Orbitrap ESI-MS. Calcd. for C_23_H_27_N_2_O_2_: 363.21, found: 364.22 [M+1], 365.22 [M+2].

For experimental use, PALs were used from stock solutions (1 mM or 4 mM) of the agent in DMSO.

### 2.3 Preparation of Aqueous Amyloid Beta

Aqueous Aβ for A11-positive oligomer generation was prepared using modifications to the protocol reported by Chunhui et al. (2018). Aβ powder was dissolved to a concentration of 2.5 mg/ml in hexa-fluoro-isopropanol (HFIP), rotated overnight and stored in 0.4 ml aliquots at −80°C. An aqueous solution was then made by combining the HFIP stock to a 2 ml microfuge tube and then, while stirring, adding 1 ml of 0.1 M NaHCO_3_, pH 9.6. HFIP was then removed by with nitrogen gas flow over the stirred solution until the volume reaches ∼0.9 ml. The sample volume was QS to 1.0 ml with 0.1 M NaHCO_3_, pH 9.6. The sample was then centrifuged at 15,000 rpm in a microfuge for 10 min to remove large amorphous aggregates as described previously (Altman et al., 2015). The supernatant was collected and the removal of large aggregates verified by circular dichroism as the broadening of the negative 200 nm band (Fezoui and Teplow, 2002; Hopping et al., 2014). The final concentration of Aβ after removal of large amorphous aggregates was ∼0.9 mg/ml (estimated from absorbance at 280 nm).

For EPR measurements, Aβ_(1–40)_ containing the TOAC spin label at position 26 (Aβ^26TOAC^) was dissolved in HFIP at 2.5 mg/ml and combined with the native peptide (2.5 mg/ml in HFIP) at a 4:1 ratio (native:TOAC-labeled). An aqueous solution of the mixture was then obtained as described above.

Samples of oligomeric Aβ (AβO) were then made by combining the 0.9 mg/ml sample of Aβ (in 0.1 M NaHCO_3_) at a 1:1 ratio with 100 mM Tris Borate (pH 7.4). After 60 min of incubation, samples were diluted to their experimental concentration with 50 mM Tris-Borate (pH 7.4) and used within 3 hours.

Aβ protofibrils were made by combining the 0.9 mg/ml sample of Aβ (in 0.1 M NaHCO_3_) at a 1:1 ratio with 100 mM Tris Borate (pH 7.0), 300 mM NaF and stirring the sample for 24-h with a 4 mm magnetic stir bar. Samples were then diluted to their experimental concentration with 50 mM Tris Borate (pH 7.0), 150 mM NaF. Beta-sheet content of protofibrils was verified by circular dichroism.

### 2.4 Cell Culture Model Over-Expressing Intracellular Amyloid Beta

MC65 cells are a neuronal cell line showing intracellular accumulation of Aβ (Jin et al., 2004; Maezawa et al., 2006; Hong et al., 2007; Maezawa et al., 2008). The MC65 cells are derived from a human neuroblastoma line with conditional expression of the carboxyl-terminal 99 residues of the amyloid-β precursor protein (APP-C99) under the negative regulation of the suppressor tetracycline (TC) in the culture medium. Expression of APPC99 is induced by removing TC from cell culture medium. Proteolysis of APP-C99 by the cellular *γ* and *β* secretases generates Aβ. Intracellular Aβ is known to start to accumulate as early as 4 h after TC removal with maximal levels at 24 h. Cell death in 3 days after removal of TC was shown to be due to the intracellular accumulation of AβO rather than to the small amounts of secreted Aβ (Maezawa et al., 2006).

The cytotoxicity was determined using MTT assay in the presence of TC, the results of which were comparable with data obtained using counts of viable cells based on trypan blue exclusion and the live/dead assay. Cells were treated with either DMSO or the indicated concentration of PAL at the same time as TC removal, with a uniform level of DMSO (0.05%) in all assay cultures. Data are expressed as mean percentage viability of 3 × 10^4^ cells/well counted from *n* = 3 cultures, with parallel +TC cultures of equal numbers of cells set at 100% viability.

### 2.5 Detection of Intracellular Oxidative Stress Signal by Confocal Microscopy

Treated and control MC65 cell cultures were gently washed with untreated culture medium and incubated for 30 min with the ROS detection reagent CellROX, a fluorogenic probe that when oxidized develops a red fluorescent signal seen around the nuclei of cells experiencing oxidative stress. At 20 min, the cells were treated for the remaining 10 min with Hoechst Blue 3342 nuclear stain, gently washed for 15 min ×3 with untreated culture medium and imaged immediately. The images of CellROX staining were collected on an Olympus Fluoview 3000 confocal laser scanning microscope. Each individual field was imaged using an 40× objective. Single plane confocal scans of the cultured neuronal cell areas were taken *via* sequential scanning mode using diode excitation lasers of 653 nm for CellROX Deep Red (λ_ex_/λ_em_ = 640/665 nm).

Intensity comparison of CellROX emission was calculated by transforming images to 8-bit gray scale and fluorescence intensity was analyzed with Image J, FIJI for MAC OS X, using the particle analysis function (Schneider et al., 2012). Triplicate measurements of the mean fluorescence intensity were done in three randomly selected areas of each of the cell culture fields, with background correction. Statistical significance between groups was determined by ordinary one-way ANOVA test using GraphPad Prism version 7.0c for MAC OS X (GraphPad Software, La Jolla California United States), where the *p* value from the ANOVA is reported as a result of the Brown-Forsythe test and considered significant if *p* < 0.05 for each treatment group. All data was expressed as the mean ±SEM.

### 2.6 A11 ELISA Assay

For ELISA measurements, peptide was passively bound to a 96-well Greiner FLUOTRAC™ 600 high binding microplate by adding 60 μl of 0.2 mg/ml aqueous Aβ solution per plate well, followed by 200 μl of freshly made 0.1 M NaHCO_3_, pH 9.6. Each assay was performed using quadruplicate wells for each sample treatment. The plates were then incubated overnight at 4°C. Wells were then treated with 300 μl blocking solution [50 mM Tris buffer (pH 7.4) containing 100 g/L dried milk] for 1-h, then washed twice with the same solution. Each well was then incubated with 300 μl of 40 μM PAL (or vehicle control) in wash buffer [50 mM Tris buffer (pH 7.4) containing 50 g/L dried milk] for 1-h, and then washed 3X with wash buffer. Wells were then treated with 300 μl of primary antibody in the presence of the PAL or vehicle control (A11 antibody diluted 1:1,200 in wash buffer containing 40 μM PAL) and incubated for 2-h. Wells were then washed 3× with wash buffer and incubated with HRP-conjugated secondary antibody (GAR antibody diluted 1:1,200 in wash buffer). Wells were then washed ×3 with wash buffer and the HRP activity quantified by luminescence by adding 150 μl of each SuperSignal (Thermo) chemiluminescent HRP substrate reagent to each well.

### 2.7 Electron Paramagnetic Resonance Spectroscopy

EPR measurements were carried out in a JEOL TE-100 X-band spectrometer fitted with a loop-gap resonator as described previously (Shea et al., 2019) (JEOL United States, Peabody, MA). PALs (or vehicle control) were added to the spin-labeled AβO (80 μM) at a final concentration of 40 μM 30 min prior to EPR measurements, carried out on ∼5 μl of sample loaded into a sealed quartz capillary tube*.* The spectra were obtained by averaging two 2-min scans with a sweep width of 100 G at a microwave power of 4 mW and modulation amplitude optimized to the natural line width of the attached spin probe. All the spectra were recorded at room temperature.

### 2.8 Circular Dichroism Measurements

For Circular dichroism spectroscopy (CD) measurements, aqueous AβΟ was diluted with 50 mM Tris-Borate, pH 7.4 to a concentration of ∼0.15 mg/ml with measurements made within 2-h. Aβ protofibrils was diluted with 50 mM Tris-Borate, 150 mM NaF, pH 7.0 to a concentration of ∼0.15 mg/ml. PALs were added from a 4 mM stock in acetonitrile to a final concentration of 40 μM. CD measurements were performed on a Jasco J-715 spectropolarimeter equipped with a Peltier temperature control (Quantum Northwest) set to 25°C. For spectral acquisition, samples were placed in a 1 mm quartz cuvette and CD spectra were collected by signal averaging three scans in the region 190–260 nm using a scan speed of 20 nm/min, bandwidth of 1 nm and response time of 4 s. Prior to analysis, all spectra were baseline-subtracted from the appropriate background buffer containing either the PAL alone or the solvent vehicle (the background signals were generally indistinguishable). The percent of secondary structure was estimated by deconvolution using the BeStSel CD analysis program (Micsonai et al., 2015), which can be accessed online at http://bestsel.elte.hu.

### 2.9 Nanoparticle Tracking Analysis

For Nanoparticle Tracking Analysis (NTA) measurements, aqueous Aβ was diluted 1:1 with 100 mM Tris-Borate, pH 7.4 to a concentration of ∼0.1 mg/ML with measurements made within 2-h. NTA was performed using a NanoSight model LM10 (Malvern Panalytical Ltd., United Kingdom), equipped with a violet (405 nm) laser and sCMOS camera. A daily calibration and data consistency confirmation was carried out using analytical standard quality polystyrene beads (Thermo Fisher Scientific, MA, United States) of sizes 70, 100, and 200 nm, and silica beads (nanoComposix, CA, United States) of sizes 80, 200, and 400 nm before the actual analyte measurements. Consequently, the samples of interest were measured by optimizing the concentration on the typical NanoSight LM10 range (∼10^8^–10^9^ particles per milliliter). Thus, typically dilutions between 1–15 k fold were applied. Filtered ultrapure Milli-Q water (resistivity = 18.2 MW cm^−1^) was used between each sample to thoroughly flush the NTA lines to confirm that the background was completely free of remnant particles before running a new sample. A 1 ml of sample was loaded into a syringe and fit into an automated syringe pump (Harvard Bioscience, MA, United States) for injection. In order to achieve rigorous and representative sampling, at minimum nine consecutive 30 s videos of each sample in flow conditions with at least 200 particle tracks present per video were recorded at camera level 12. The data was analyzed using NanoSight NTA 3.1 software with the detection threshold set to 5 and screen gain 10 to track the statistically relevant number of particles, simultaneously minimizing the distorting background artifacts.

### 2.10 Thioflavin T Assay

Three microliters of aqueous Aβ (∼0.9 mg/ml in NaHCO_3_) were added to the wells of a black, Nunc MicroWell, 384-well nonbinding optical bottom microplate (cat # P9241-30EA) containing 50 μl of PBS. Samples were treated with PALs (18 μM) or vehicle control. Prior to each assay, a fresh 1 mM ThT (Sigma Aldrich, product # T3516) was prepared in cold DI water and filtered through a 0.22 µm syringe filter. Assays were initiated with 1 μl of ThT. ThT fluorescence was measured at room temperature, using a TECAN Infinite 200Pro plate reader, through the bottom of the plate, with of 440 nm and emission of 486 nm. The fluorescence intensity of a free ThT solution (20 µM) in PBS was used for background subtraction of the control sample and the background of ThT + PAL was subtracted from samples containing Aβ.

### 2.11 Nile Red Assay

Three microliters of aqueous Aβ (∼0.9 mg/ml in NaHCO_3_) were added to the wells of a black, Nunc MicroWell, 384-well nonbinding optical bottom microplate (cat # P9241-30EA) containing 50 μl of PBS. Samples were treated with PALs (18 μM) or vehicle control. Prior to each assay, a fresh solution 1 mM Nile Red (Sigma Aldrich, product # 19123) was prepared in DMSO and centrifuged at 10,000 RPM to remove any aggregates. Assays were initiated with 1 μl of Nile Red. Nile Red fluorescence at *t* = 5 h was measured at room temperature, using a TECAN Infinite 200Pro plate reader, through the bottom of the plate, with of 558 nm and emission of 635 nm. The fluorescence intensity of a free Nile Red solution (20 µM) in PBS was used for background subtraction of the control sample and the background of Nile Red + PAL was subtracted from samples containing Aβ.

### 2.12 Cytokine Measurements

#### 2.12.1 Human TGRL Isolation

The protocol for obtaining human TGRL (Protocol No. 447043) was approved by the Human Subjects Review Committee/IRB at the University of California Davis and informed consents were obtained from all study subjects. Healthy adult human volunteers consumed a moderately high-fat meal containing at least 40% fat, and postprandial (3.5 h) blood was collected by standard venipuncture (Vacutainer K2EDTA tubes; BD, Franklin Lakes, NJ). We recruited five to six human donors/week, pooled the plasma, and isolated TGRLs. The average pooled TGRL concentration was ∼700–800 mg/dl. We used 150 mg/dl concentration to treat endothelial cells in our study. For experiments, we pooled TGRL isolated from donors. Through extensive experience, we have found the data were very consistent using this method of collection and pooling of TGRLs (Aung et al., 2013; Aung et al., 2014; Aung et al., 2016). Whole blood samples were then centrifuged at 3,000 rpm for 15 min at 4°C, and the plasma fraction was collected. Sodium azide was added to the plasma as a preservative. TGRL were isolated from human plasma at a density of less than 1.0063 g/ml following an 18 h centrifugation at 40,000 rpm in a SW41 Ti swinging bucket rotor (Beckman Coulter, Sunnyvale, CA, United States) held at 14°C within a Beckman L8-70M (Beckman) ultracentrifuge. The top fraction TGRL was collected and dialyzed in Spectrapor membrane tubing (MWCO 3,500; Spectrum Medical Industries, Los Angeles, CA, United States) at 4°C overnight against a saline solution containing 0.01% EDTA. Total triglyceride content of samples was determined using the serum triglyceride determination kit (Sigma Aldrich cat # TR0100). The kit converts triglycerides to free fatty acids and glycerol. Glycerol is assayed enzymatically.

#### 2.12.2 Cell Culture and Lipid Treatments

Human brain microvascular endothelial cells (HBMECs) were obtained from Angio-Proteome (Boston, MA, United States) and cultured in EGM™-2MV BulletKit™ containing 5% serum (CC-3202, Lonza, Walkersville MD) in a 37°C incubator with a humidified 5% CO_2_ and 95% air environment. Medium was changed every other day until 90% confluency and cells were used at passage 6. One hour prior to experiments, cell culture medium was changed to fresh medium. Cells were exposed for 3 h to the following conditions: control of media containing DMSO at a final concentration of 0.01% (control) and TGRL hydrolyzed with lipoprotein lipase (L2254, Sigma, St. Louis, MO, United States) [referred to as TGRL lipolysis product, TGRL (150 mg/dl = 1.5 mg/ml) + lipoprotein lipase (LpL; 2 U/mL)]. The final concentration of TGRL lipolysis products were diluted in media and pre-incubated for 30 min at 37°C prior to application. After the incubation with media or TGRL lipolysis products, cells were washed with cold PBS and harvested by scraping them in ice cold PBS.

To test the suppression of compounds on TGRL lipolysis products-induced gene expression, cells were pre-incubated with each individual compound of interest indicated for 30 min and followed by co-incubated with TGRL lipolysis for 3 h. These compounds are Tempo, Mito-Tempo, PMT-301, PMT-302, PMT-303, PMT-401, and PMT-402. The final concentration of each compound is 1 µM. After the incubation, the cells were washed with cold PBS and mRNA expression of ATF3, E-selectin, IL-8, IL-6, and COX-2 were analyzed.

#### 2.12.3 mRNA Expression by Quantitative RT-PCR

Total RNA was extracted from cells in each of treatment group [control or TGRL lipolysis products (TL) or TL + individual compound of PAL] in 6-well plate (3 well per sample, *n* = 3/group) using RNeasy Mini Kit (Qiagen, Valencia, CA, United States) including the DNA digestion step as described by the manufacturer. Sample quality was assessed using Nanodrop ND-1000 Spectrophotometer (Thermo Fisher Scientific, Wilmington, DE, United States). An aliquot equivalent to 5 μg of total RNA extracted from each sample was reverse-transcribed to obtain cDNA in a final volume of 21 μl consisting of buffer, random hexamers, DTT, dNTPs, and SuperScript^®^ III First-Strand Synthesis System (Invitrogen). qRT-PCR with SYBR as fluorescent reporter was used to quantify the gene expression. Specific human primers were designed with Primer Express 1.0 software (Applied Biosystems) using the gene sequences (Supplementary Table S1) obtained from previously published Affymetrix Probeset IDs (Aung et al., 2014). Reactions were carried out in 384-well optical plates containing 25 ng RNA in each well. The quantity of applied RNA was normalized by simultaneously amplifying cDNA samples with glyceraldehyde-3-phosphate dehydrogenase (GAPDH)-specific primers. The transcript levels were measured by real-time RT-PCR using the ABI ViiA™7 Real-Time PCR system (PE Applied Biosystems, Foster City, CA, United States). The PCR amplification parameters were: initial denaturation step at 95°C for 10 min followed by 40 cycles, each at 95°C for 15 s (melting) and 60°C for 1 min (annealing and extension). A comparative threshold cycle (Ct) method was used to calculate relative changes in gene transcription determined from real-time quantitative PCR experiments [Applied Biosystems user bulletin no. 2 (P/N4303859)] (Livak and Schmittgen, 2001). The threshold cycle, Ct, which correlates inversely with the target mRNA levels, was measured as the cycle number at which the SYBR Green emission increases above a preset threshold level. The specific mRNA transcripts were expressed as fold difference in the transcription of the specific mRNAs in RNA samples from the TL or TL + individual compound of PAL (Tempo or Mito-Tempo or PMT-401or PMT-402 or PMT-101 or PMT-301 or PMT-302 or PMT-303)-treated cells compared with those from the control-treated cells.

#### 3.12.4 Statistical Analysis

Data for changes in gene expression obtained by qRT-PCR were analyzed by GraphPad PRISM software (San Diego, CA). An unpaired student’s t test was used for comparisons between treatments. Differences with *p* ≤ 0.05 were considered significant. Results are expressed as MEAN ± SEM.

## 3 Results

### 3.1 Differential Potency of Stilbene Paramagnetic Amyloid Ligands

**FIGURE 1:**
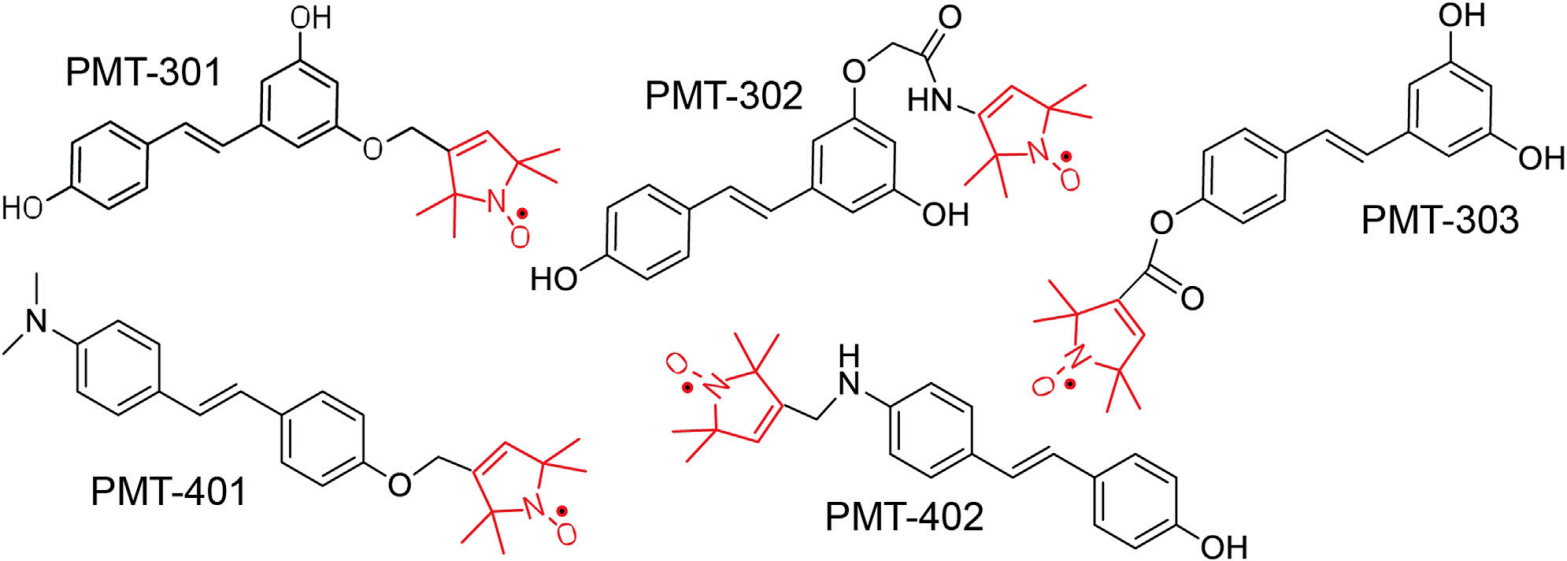
Structure of Paramagnetic Amyloid Ligand (PAL) candidates. The stilbene core binds to oligomeric Aβ, inhibiting oligomer growth and toxicity. The spin label moiety (red) contains the nitroxide that functions as a catalytic antioxidant.

**FIGURE 2:**
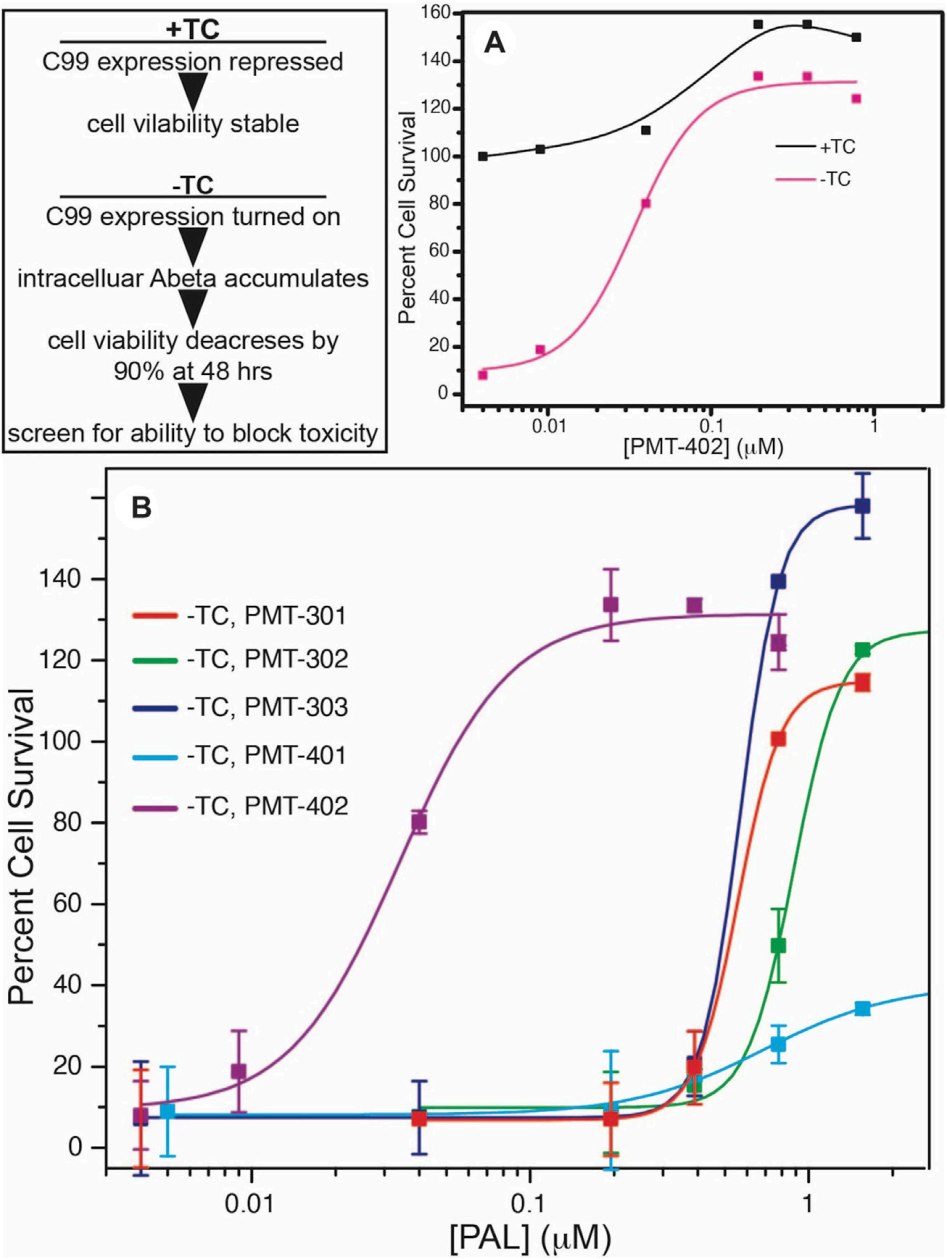
Neuronal protection activity of the stilbene PAL agents in the MC65 model. Removal or tetracycline from the MC65 culture media results in C99 expression and amyloid beta cytotoxicity **(A). (B)** shows rescue of cytotoxicity with PAL titration. Error bars are the SEM from the assay of three separate cultures.

The structures of the stilbene-based PALs are shown in Figure 1. Importantly, these results demonstrate the utility of the MC65 model in measuring intracellular AD pathology such as oxidative stress (Hilt et al., 2018), Ca^2+^-dysregulation (Copenhaver et al., 2011), activation of inflammatory pathways (Currais et al., 2016) as well as autophagy and pTau formation (Mputhia et al., 2019). In the MC65 model, expression of the progenitor of the Aβ peptide (C99) is repressed in the presence of tetracycline (+TC). Upon removal of tetracycline (−TC), induction of C99/AβO decreases the cell viability by ∼90%. The ability of the stilbene PALs to protect against Aβ-induced cytotoxicity was assessed using the MC65 neuronal culture model, where the inducible expression of the C99 fragment of APP results in cell death within 72 h (Petrlova et al., 2012; Currais et al., 2016). Each of the stilbene PALs provides protection against cell death in the MC65 assay (Figure 2). However, the potency of the PAL candidates varies, with PMT-402 more than 50-times more potent than PMT-302. PMT-401 was the only PAL that could not restore viability to 100%. The poor performance of PMT-401 at higher concentrations is likely related to its poor solubility.

### 3.2 Stilbene Paramagnetic Amyloid Ligands Reduce Binding of the Conformation-Specific Antibody A11

**FIGURE 3:**
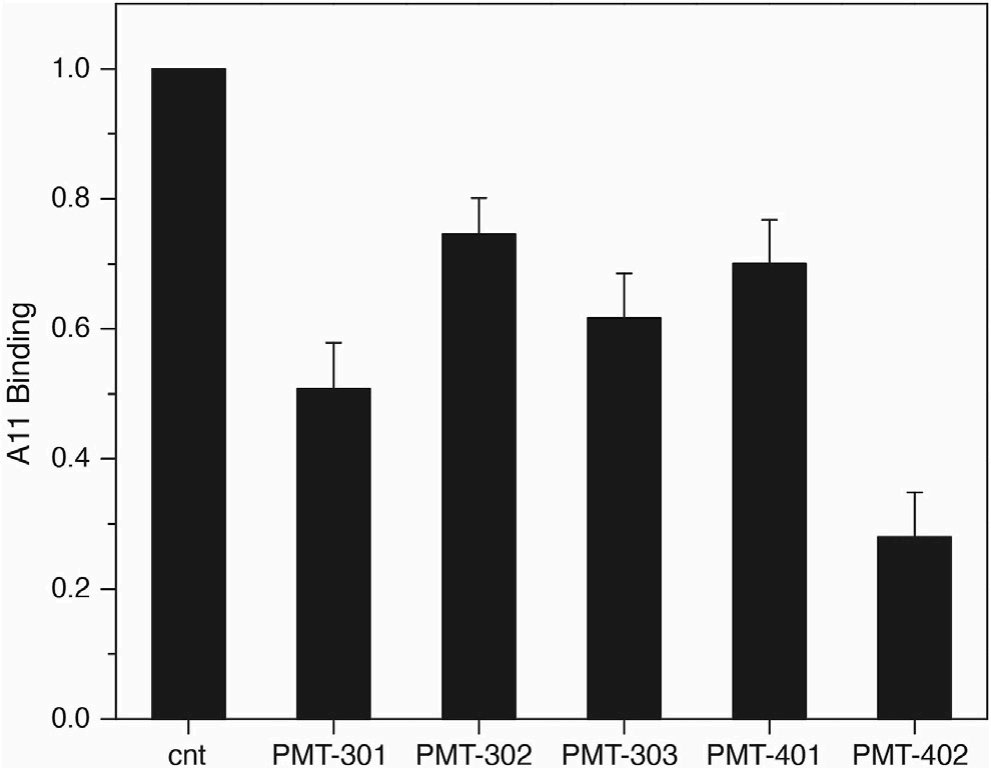
Decrease in A11 antibody recognition of AβO following PAL treatment. the A11 antibody. Shown is the oxidized luminol signal (normalized to the untreated control) as reported by the HRP-secondary antibody. Quadruplicate samples were measured in each assay. Results are the average of three independent assays with error reported as SEM.

The identification by Glabe and co-workers (Kayed et al., 2003) of an antibody that recognizes the pathogenic state of disparate proteins involved in neurodegeneration provides a tool for probing modulation of the AβO away from its “toxic” conformation (Necula et al., 2007). To evaluate the effect of the stilbene PAL agents on A11 recognition, we measured A11 binding to immobilized AβO with and without PAL treatment. As shown in Figure 3, treating immobilized AβO with a stoichiometric amount of the PAL agent reduces the average A11 capture by ∼50%. PMT-402 appears most effective in reducing the amount of A11 recognition in this assay.

### 3.3 Paramagnetic Amyloid Ligands Combat Aβ-dependent Oxidative Stress

**FIGURE 4:**
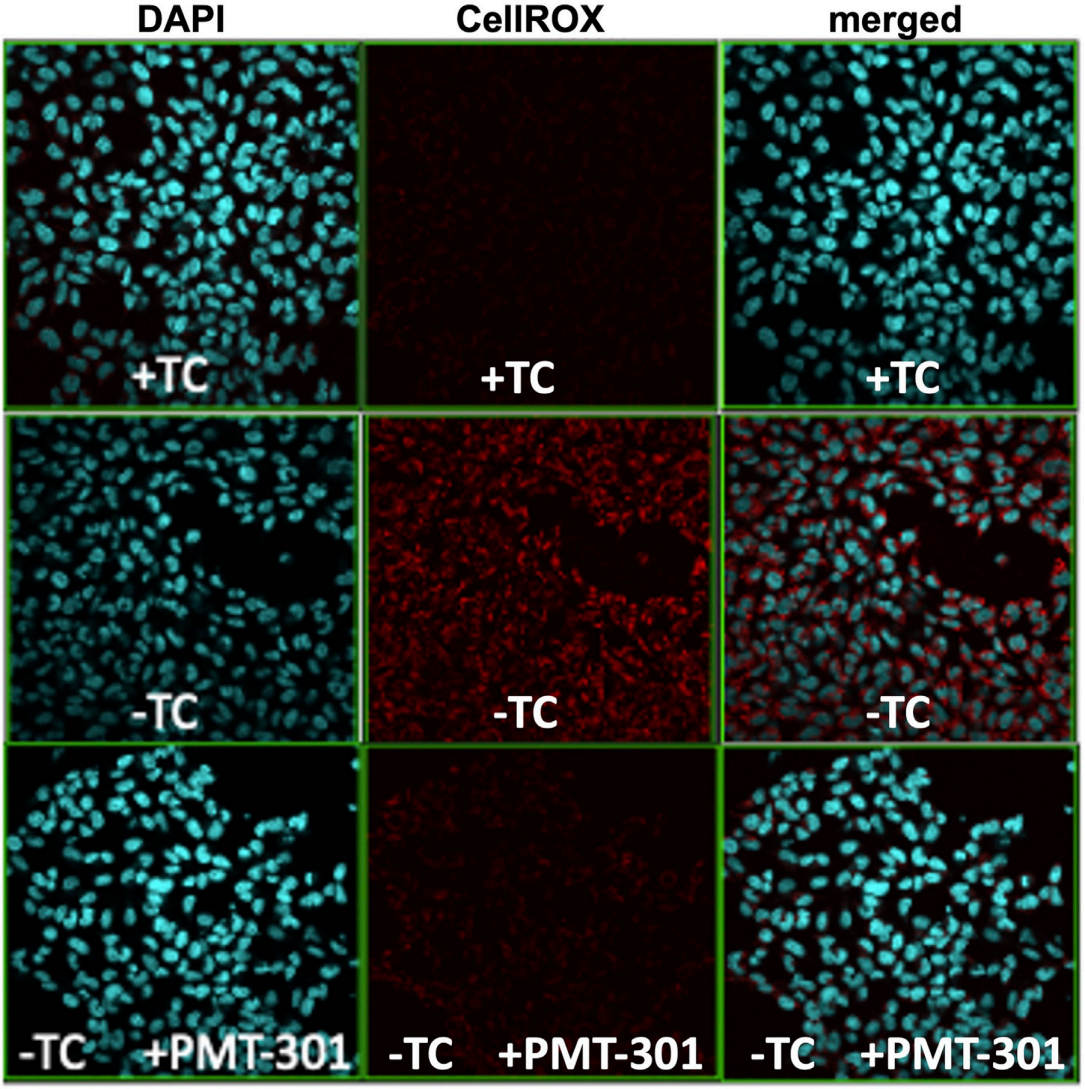
Confocal imaging of the CellRox dye to measure the PAL reduction of oxidative stress. Induction of C99/Ab (−TC) results in ROS production (red punctae in second row, center panel). PMT-301 reduces oxidative stress levels in MC65 cells (third row, center panel) to levels comparative to +TC protected state.

We have previously demonstrated the highly potent antioxidant capacity of the nitroxyl-based PALs (Hilt et al., 2018). To confirm this property in the stilbene PALs, the CellROX dye in MC65 cells was imaged for levels of reactive oxygen (ROS) species. Figure 4 illustrates the antioxidant activity of the PMT-301 PAL. Shown are confocal microscopy images of the ROS-sensitive CellROX dye (red) in live MC65 neurons. Oxidative stress is absent when expression of the progenitor of the Aβ peptide (C99) is repressed (+TC, top row). Induction of C99/Aβ production (−TC) generates high ROS levels (middle row). In contrast, as shown in the bottom row, ROS levels in −TC cells are highly (∼60%) attenuated by PMT-301 treatment (see Supplementary Figure S6).

Oxidative-stress and inflammation are closely connected in neurodegenerative disorders (Bonda et al., 2010; Liu et al., 2017), so the PAL antioxidant activity is expected to also attenuate markers of inflammation. In order to access whether the PALs elicit anti-inflammatory activity independent of the AβO-driven oxidative stress, we also looked at whether any of the stilbene PALs carry anti-inflammatory activity in the human brain endothelial cell (HBMEC) model (Aung et al., 2014). In this model, TGRL lipolysis products (TL) are used to upregulate stress-responsive transcription factor ATF3, COX-2, and proinflammatory genes (IL-6, IL-8 and E-selectin) using microarray data analysis (Aung et al., 2014) and RNA Seq analysis (Nyunt et al., 2019). TL substantially increases stress-responsive transcription factor ATF3, COX-2, and proinflammatory genes (IL-6, IL-8 and E-selectin). We previously showed that TL causes lipotoxic injury to HBMECs and this lipotoxicity occurs through stimulation of mitochondrial metabolism resulting in overproduction of superoxide radical (O_2_•^−^) (Aung et al., 2016). Moreover, TL increased mitochondrial O_2_•^−^ generation, ATF3-mediated inflammatory, and apoptotic responses in *in vitro* HBMECs culture (Nyunt et al., 2019). Here we analyzed the biological activity of the stilbene PALs plus two common nitroxide agents (Tempo and Mito-Tempo) on TL-induced gene expression. As a group, only PMT-401 provides broad anti-inflammatory activity as measured by gene expression. As shown in Supplementary Figure S7, PMT-401 significantly suppressed TL-induced ATF3, E-selectin, IL-6, IL8, and COX-2 gene expression. Additionally, COX-2 expression was suppressed by PMT-302 and PMT-303 (*p* = 0.07). Because PMT-401 is distinguished in part by its greater hydrophobicity (CLogP of 4.5, vs. CLogP values of 2.9–4.0 for the other PAL compounds), the effectiveness of nitroxide antioxidants to attenuate inflammation in the TL-activated HMMEC model may rely on the compound’s ability to partition into a lipophilic environment. Consistent with this notion is the lack of anti-inflammatory activity found with Tempo and Mito-Tempo treatment, and in fact increased TL-induced ATF3, E-selectin, IL-6, IL8 and COX-2 gene expression. Thus, the simple addition of a hydrophilic nitroxide is insufficient to attenuate inflammation in this model.

### 3.4 Target Engagement and Conformational Adaptation

**FIGURE 5:**
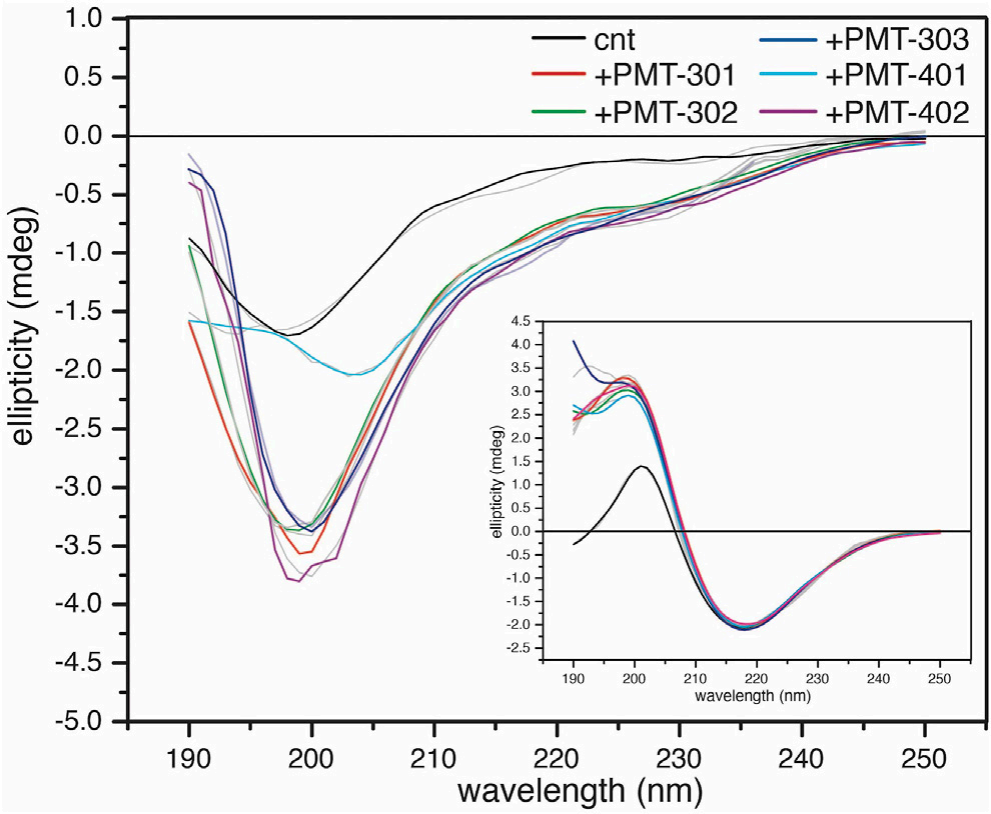
CD spectra of AβOs treated with a stoichiometric amount of PAL agent. CD spectra of 1-h AβO treated with 40 μM PAL. The control (black) was treated with an equal volume of vehicle. The inset shows the high β-sheet structure fibrillar oligomers show little structural response to the PAL agents. BeStSel fits of the spectra are given in light gray lines.

In order to investigate how the stilbene PALs affect AβO conformation, CD spectroscopy was carried out to probe for secondary structure changes in the PAL-treated peptide. The untreated early AβO generates a low amplitude CD spectrum indicative of its unstructured state (Shea et al., 2019; Clements et al., 1996) (Figure 5, black trace). After 24 h, the AβO sample undergoes a substantial increase in the pleated beta-sheet content as it converts into soluble protofibrils (Walsh et al., 1999; Kayed et al., 2009) (Figure 5, inset). While each of the stilbene PALs induce significant changes to the CD spectrum of AβO, the stilbene PALs do not order the early AβO into an α-helical or β-strand state. This finding is consistent with previous CD measurements of stilbene effects on AβO (Ladiwala et al., 2010; Feng et al., 2009). Nevertheless, each of the five PALs drive a significant change in the AβO CD spectrum, with PMT-301, PMT-302, PMT-303 and PMT-402 generating strong negative bands at 198 nm, characteristic of PP-II structure (Adzhubei et al., 2013). This response is similar to a fluorene-based PAL (Altman et al., 2015). In contrast, PMT-401 does not induce the PPII-like spectral change. Results from the fitting of the CD spectra by the BeStSel algorithm (Micsonai et al., 2015) to estimate fractions of secondary structure are given in Table 1. In each of the early AβO samples, the low structure folds (random coil/loop, 3_10_- and π-helices, PP-II helix) constitute the major population before and after PAL treatment. Of the compounds that produce a PPII-like spectrum, only a marginal increase in α-helical structure is seen, accompanied by a slight decrease in the β-strand population. The effect of PMT-401 on AβO is distinguished by helical content and decreased beta content compared to the other PALs. Interestingly, PMT-401 (which displays the lowest potency in cell protection) generates a very unique CD spectrum for AβΟ in an aqueous solution, although a similar 204 nm minima has been reported following treatment of AβO with 10% trifluoroethanol or 50% acetonitrile (Fezoui and Teplow, 2002; Bartolini et al., 2007).

**TABLE 1:** Results from the fitting of the CD spectra by the BeStSel algorithm to estimate fractions of secondary structure in Aβ samples.

Species	AβO						Protofibril Aβ					
	cnt	301	302	303	401	402	Cnt	301	302	303	401	402
α	0	4.5	2.6	2.8	7.2	2.8	1.9	0	0	0	0	0
β	40.9	34.6	38.6	35.2	30.4	35.0	50.3	57.0	57.2	60.6	57.1	57.3
Turn	14.4	15.6	14.7	15.0	15.7	15.4	10.6	9.6	10.3	11.3	10.7	10.7
Other	44.7	45.3	44.2	47.0	47.1	46.7	37.2	33.4	32.5	28.1	32.1	32.1

Finally, we also looked to see the effect of PAL addition to protofibrillar oligomers formed after 24-h incubation (Figure 5, inset). These species have much lower toxicity and lack recognition by the conformational antibody A11 (Kayed et al., 2009; Bieschke et al., 2011). Consistent with previous findings (Walsh et al., 1999; Bartolini et al., 2007; Corsale et al., 2012; Roychaudhuri et al., 2014), the CD spectrum of the protofibril Aβ sample displays a high percentage of β-strand. In this more ordered species, the CD spectra of each PAL-treated preparation is similar to the untreated control, although each of the PALs enhance the conversion of the disordered components into β-strand by 7%–10% (Table 1).

### 3.5 Alteration of Amyloid Beta Oligomers Dye Binding

**FIGURE 6:**
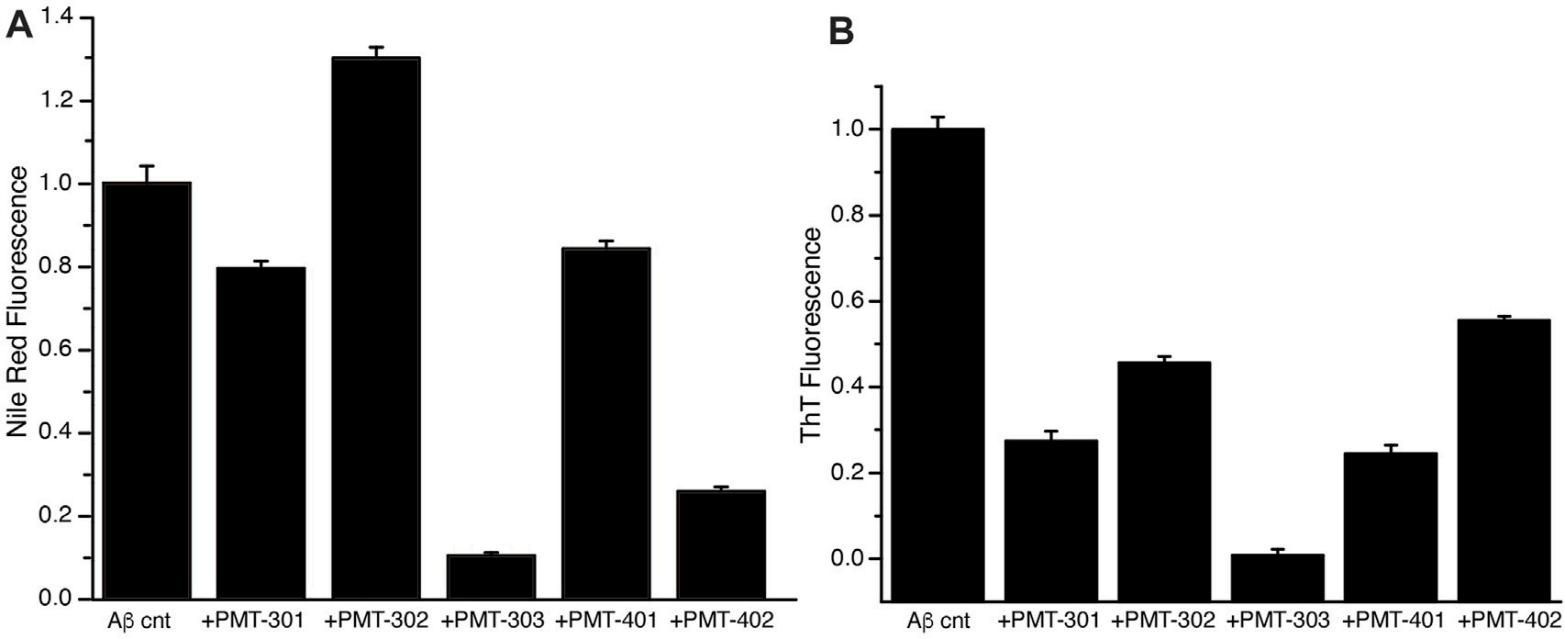
PAL effect on AβO-associated dyes. **(A)** Nile Red fluorescence intensity with or without designated PAL addition after 5-h incubation. **(B)** ThT fluorescence intensity at *t* = 24 h was measured for Aβ alone or Aβ + PAL compounds. All samples were run in triplicates.

We also measured the effect of the stilbene PALs on modulating the interaction of dyes with AβO over time, whose fluorescence is influenced by its association with proteins and their aggregates. The fluorescence of the Nile Red (NR) dye is indicative for its degree of solvation, where its quantum yield increases within more hydrophobic environments (Singh et al., 2013). The NR fluorescence intensity in the presence of AβO with and without PAL treatment is shown in Figure 6A. Each of the stilbene PALs, except for PMT-302, increases the solvent exposure of NR. We also looked at the fluorescence intensity of Thioflavin T (ThT), which is greatly amplified upon its intercalation within β-sheet structure. ThT fluorescence therefore provides a fundamental marker for Aβ assembly along the amyloidogenic pathway (Hellstrand et al., 2010). To evaluate the ability of the stilbene PALs to inhibit AβO conversion into a protofibril species, ThT fluorescence in the presence of AβO was measured with and without PAL treatment. Figure 6B shows the ThT signal following 24-h of incubation. These results, in combination with the NR findings, suggest PMT-303 is highly efficient in both AβO conformational adaption and inhibition of β-sheet formation.

### 3.6 Effect of Paramagnetic Amyloid Ligands on Amyloid Beta Oligomers Structure

**FIGURE 7:**
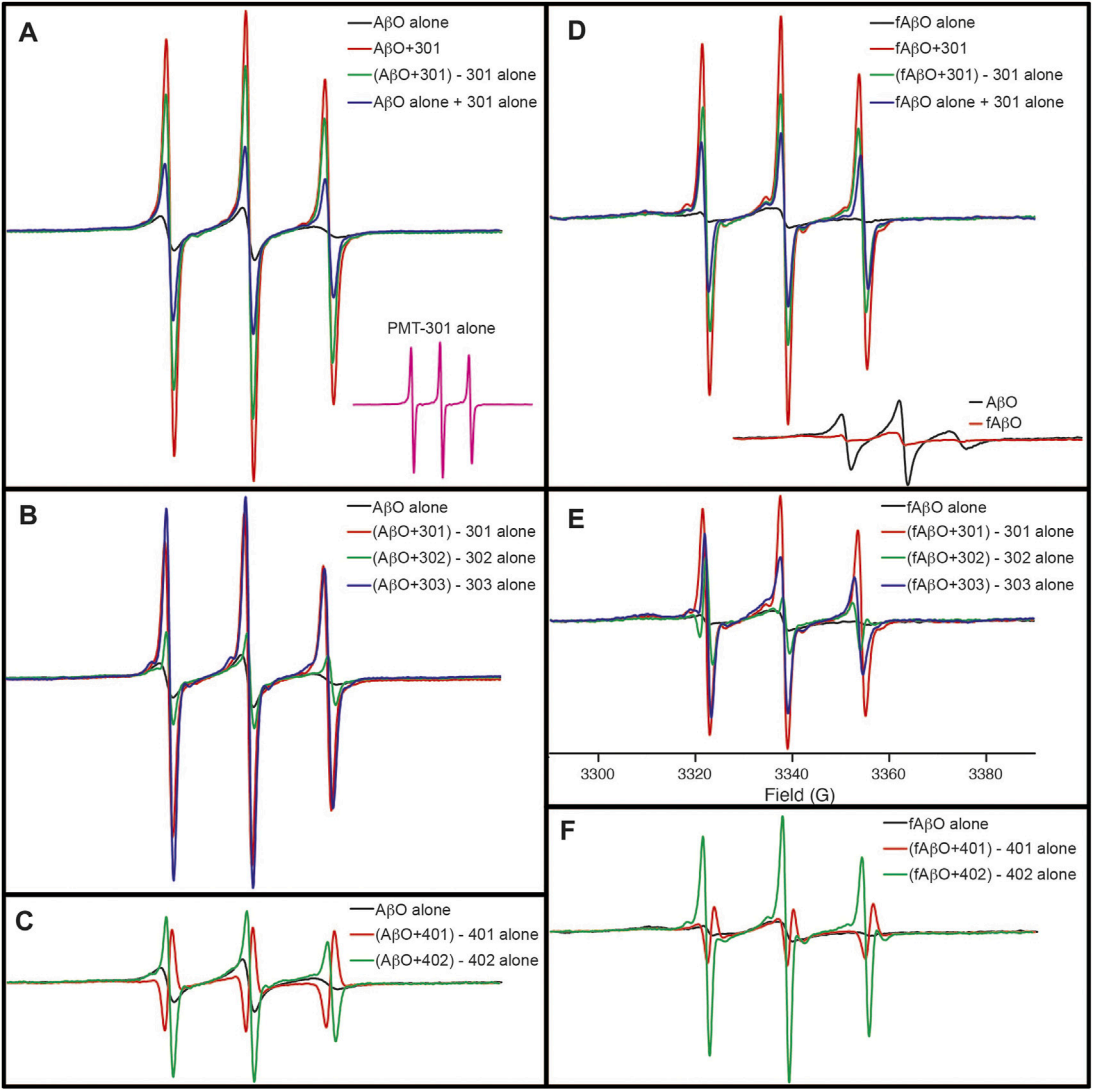
EPR spectra showing the how PALs alter the order within AβO. Shown are X-band EPR spectra of AβO^TOAC^ with and without PAL treatment. The black trace in panel **(A)** shows the broad spectrum of the TOAC label within AβO, reflecting a local region of order and a close proximity to other TOAC labels. In contrast, the EPR spectrum of the PMT301 PAL alone displays a narrow line shape owing to the rapid rotational motion of the free small molecule in solution [**(A)**, inset]. Upon addition of PMT301 to AβO, a composite spectrum is obtained [red trace in **(A)**]. The effect of the PAL agent on the isolated AβO signal can be observed by subtracting the spectrum of free PMT301 from the composite AβO + PMT301 spectrum to produce the resulting PAL-modified AβO spectrum [green trace in **(A)**]. The increased amplitude of the green spectrum in panel A reflects a decrease in the TOAC spin label order following PMT301 treatment. The conformational effect of PMT301 is also evident when comparing the experimental composite spectrum (red trace) to the calculated sum of the two samples alone [blue trace in panel **(A)**]. **(B,C)** show the comparative effects of each PAL on AβO. In **(B,C)** the untreated AβO spectrum (black trace) is compared to the sample after subtraction of the PAL-alone (free PAL) component. **(D–F)** show the results of similar measurements on the protofibril AβO (fAβO) sample. A comparison of AβO to fAβO is shown in the inset of **(D)**. fAβO displays a broader EPR spectrum than AβO, reflecting the increased order and spin coupling found in the protofibril sample. Notably, subtraction of the free PMT401 component from the composite AβO and fAβO spectra results in spectra with inverted amplitudes [red traces in **(C,F)**]. This finding reveals evidence of spin coupling between the PMT401 nitroxyl moiety and the TOAC label on Aβ (i.e., the PMT401 contribution is substantially broadened in both the composite AβO + 401 spectrum and fAβO + 401 spectrum. Except for the inset spectra in **(A,D)**, spectral intensities represent stoichiometric amounts of AβO^TOAC^ and PAL agent (both at 80 μM). The spectral amplitudes of each panel are normalized to the same amount of AβO^TOAC^, and all spectra were collected over 100 G [field sweep axis displayed in **(E)**].

Although the CD results do not indicate conversion into a well-defined state of secondary structure, electron paramagnetic resonance (EPR) spectroscopy of AβO’s reveals, that with the exception of PMT-401, the stilbene PALs trigger a major reorganization of the peptide’s central region. In these experiments, Ser26 of Aβ was substituted with TOAC, an amino acid spin label. The TOAC nitroxide provides a sensitive indicator of both the local within the peptide and its proximity to other spin labels within the assembly (Petrlova et al., 2012; Altman et al., 2015). Thus, although CD spectroscopy reveals a lack of secondary structural order in the AβOs, the central turn region provides a constricted environment at position 26, and represents a common region for peptide-peptide interaction. The level of spin-coupling in the sample was attenuated by preparing AβOs consisting of 75% native Aβ and 25% of the TOAC-substituted Aβ (AβO^TOAC^). The EPR spectra of the PAL-treated AβO^TOAC^ are shown in Figure 7. The black trace in panel A shows the broad spectrum of the TOAC label within AβO, reflecting a local region of order and a close proximity to other TOAC labels. This indicates the central hairpin turn region facilitates self-interaction within the oligomer. In contrast, the EPR spectrum of the PMT301 PAL alone displays a narrow line shape owing to the rapid rotational motion of the free small molecule in solution (panel A, inset). Upon addition of PMT301 to AβO^TOAC^, a composite spectrum is obtained (red trace in panel A). The effect of the PAL agent on the isolated AβO^TOAC^ signal can be observed by subtracting the spectrum of free PMT301 from the composite AβO^TOAC^ + PMT301 spectrum to produce the resulting PAL-modified AβO^TOAC^ spectrum (green trace in panel A). Thus, by comparing the black trace to the green trace we are comparing the AβO central region dynamics before and after PMT301 treatment. The altered state of the TOAC can also be confirmed by comparing the experimental composite spectrum (red trace) to the calculated sum of the two samples alone (blue trace in panel A). For example, if the PAL had no effect on AβO dynamics, the red and the blue traces would be identical. Panels B and C show the comparative effects of each PAL on AβO^TOAC^. Here, the untreated AβO^TOAC^ spectrum (black trace) is compared to the sample after the PAL-alone component was subtracted. Thus, comparing each sample to the untreated AβO (black trace) reveals the degree of alteration each PAL has on the oligomer’s central region. These results show that PMT301, PMT303 and PMT402 are most effective in increasing the dynamics at position 26 of Aβ. This is a significant finding, as PALs provide the highest potency in protection against AβO toxicity. In contrast the PMT302 treated AβO spectrum (panel B, green trace) looks similar to control AβO (panel B, black trace). Remarkably, the compound with the lowest potency (PMT401), is not only incapable of increasing the local dynamics of the turn region but displays spin-spin interaction with the TOAC label. This is evident from the resulting inverted amplitude line shape following subtraction of the free PMT401 component from the composite AβO (panel C, red trace). This indicates PMT401 binds and maintains AβO in a stable conformation with its nitroxyl moiety close (<1 nm) to the vicinity of TOAC26.

The effect of the stilbene PALs on the protofibril AβO^TOAC^ was also explored (Figures 7D–F). Panels D-F show the results of similar measurements on the protofibril AβO (fAβO) sample. A comparison of AβO to fAβO is shown in the inset of panel D. fAβO displays a broader EPR spectrum than AβO (including a discernable strongly immobilized component), reflecting the increased order and spin coupling found in the protofibril sample (Petrlova et al., 2012). The PALs have a similar, though smaller effect on fAβO. Thus, although CD reveals that PALs are unable to reverse the beta structure in the protofibril species, with the exception of PMT401, they do affect the packing geometry about the central region of the peptide.

### 3.7 Nanoparticle Tracking Analysis Measurements of Amyloid Beta Oligomers Treated With Each Stilbene PAL Candidate

**FIGURE 8:**
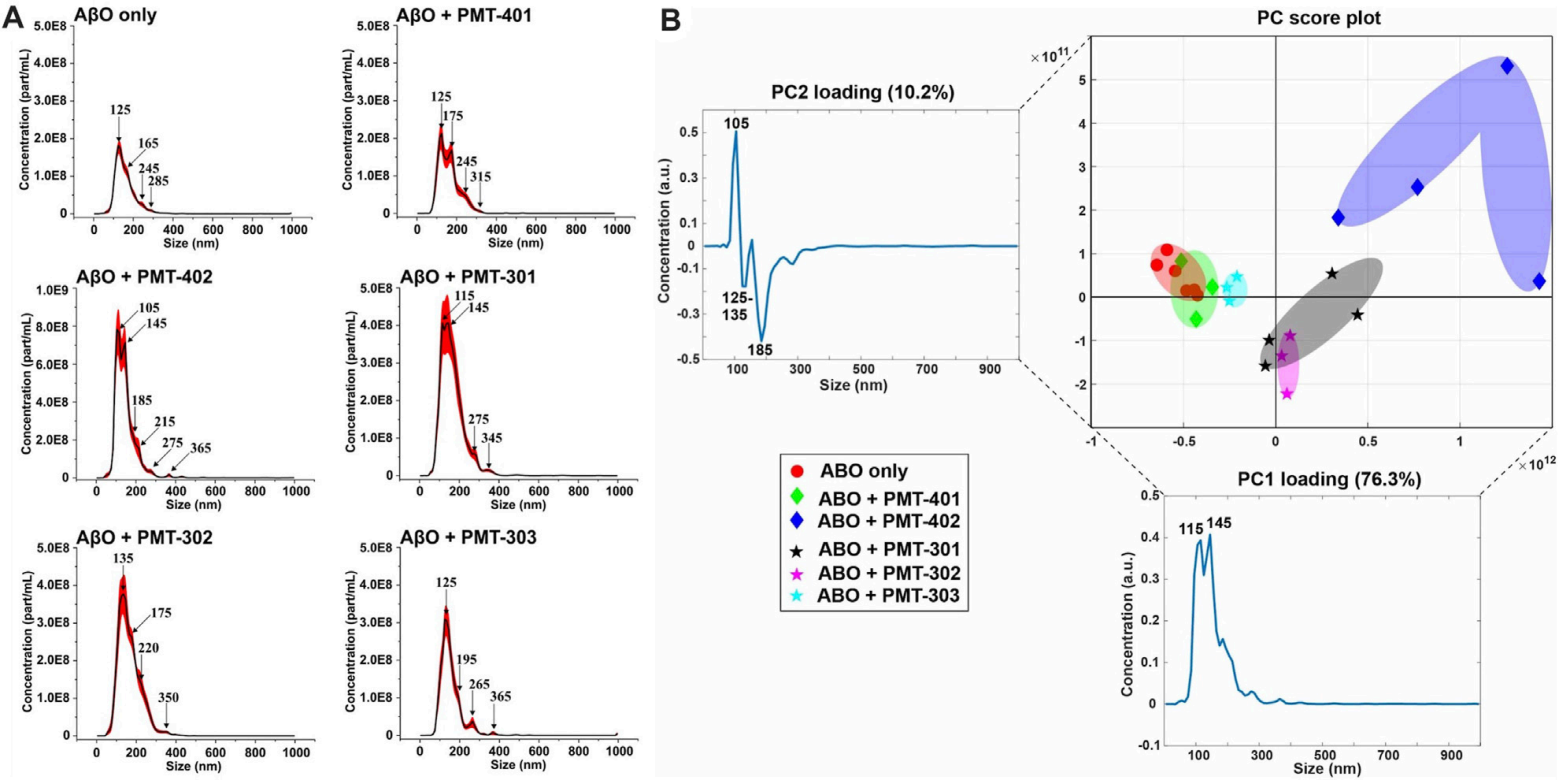
NTA measurements of AβO treated with each stilbene PAL candidate. **(A)** Nanoparticle Tracking Analysis (NTA) scattering profiles of AβO preparations with and without PAL treatment. The black curves represent the average particle size vs. concentration distribution recorded and analyzed from 9–18 × 30 s videos and the red curves display the corresponding ±1 standard deviations. **(B)** A principal component analysis (PCA) on the NTA size distribution data for different combinations of AβO and stilbene PALs. The 2-dimensional PC score plot displays the separation of the tested groups: AβO alone (red circles) from a total of 1.23 × 10^5^ particles detected and analyzed and on average 6.86 × 10^3^ particles per video detected and analyzed); AβO + PMT-401 (green diamonds) from a total of 3.56 × 10^4^ particles and on average 3.95 × 10^3^ particles; AβO + PMT-402 (blue diamonds) from a total of 6.26 × 10^4^ particles and on average 5.22 × 10^3^ particles; AβO + PMT-301 (black stars) from a total of 1.35 × 10^5^ particles and on average 7.51 × 10^3^ particles; AβO + PMT-302 (magenta stars) from a total of 7.19 × 10^4^ particles and on average 7.98 × 10^3^ particles; AβO + PMT303 (cyan stars) from a total of 4.40 × 10^4^ particles and on average 4.89 × 10^3^ particles. The PC1 loading explains 76.3% of the total variation in the examined data set whereas PC2 loading corresponds to 10.2% of the total variation. Two prominent peaks can be distinguished in PC1 loading spectrum at 115 and 145 nm, and respectively PC2 reveals three predominant peaks at 105 nm, 125–135 nm, and 185 nm. In the PC score plot (upper right), the shaded areas serve as visual guides to discern the different groups.

We examined AβO preparations by Nanoparticle Tracking Analysis (NTA). NTA utilizes the properties of both light scattering and Brownian motion in order to obtain the nanoparticle size distribution of samples in liquid suspension. Briefly, 9–18 videos of 30 s duration per each sample were acquired, with a frame rate of 30 frames per second. The NTA software is optimized to first identify and subsequently track each particle on a frame-by-frame basis. The velocity of particle movement is used to calculate particle size by employing the two-dimensional Stokes-Einstein equation. Given the relatively low refractive index of protein/peptide species, the smallest detectable size using the NTA system is roughly in the order of 70 nm. Since volume of the sample chamber is known and the NTA is essentially a single-nanoparticle detection system whereby each detected particle in the field of view over the duration of the recorded videos is calculated, the instrument also yields concentration data as particles per milliliter (part/ml). Thus, the data consists of particle concentration on the ordinate (y, part/ml) as the function of detected particle size on the abscissa (x, nm). The AβO prep is expected to generate a broad range of sizes (Hepler et al., 2006; Corsale et al., 2012), ranging from oligomers of 50–200 kD to much larger amylospheroid and protofibrils, which do not migrate through size exclusion. For these measurements, stoichiometric amounts of PALs were added to preformed AβOs. The NTA results shown in Figure 8A indicate that the PAL compounds do not dramatically change the observed particle size, however each PAL generates a distinct distribution. There is a moderate correlation between the width of the major distribution peak to neuronal protection, with a narrower distribution favoring better protection. For example, the full-width at half maximal value (FWHM) of the distributions to potency of neuronal protection, with two of the more potent PALs (PMT-303 and PMT-402) having FWHM values ∼45% lower than the other agents (Table 2). A major advantage of the NTA measurements is their ability to quantify the particle concentration. As shown in Table 2, each of the PALs increases the population (relative to the untreated control) of oligomers into the NTA observable regime. Thus, a depletion of the smaller more toxic oligomers may constitute a part of the PALs’ protective mechanism.

**TABLE 2:** Calculated particle parameters from NTA analysis of AβO samples.

Specimen	Average size (nm)	Mode size (nm)	Conc (part/ml)	195–595 nm fraction (%)	FWHM
ABO Only	159.2 ± 1.5	127.9 ± 3.3	1.59 ± 10^9^ ± 3.70 ± 10^7^	20.3	76.58
ABO + PMT-401	161.8 ± 1.9	124.1 ± 6.3	2.07 ± 10^9^ ± 9.12 ± 10^8^	23.7	90.44
ABO + PMT-402	145.9 ± 2.2	116.7 ± 3.7	3.40 ± 10^9^ ± 1.10 ± 10^8^	28.7	71.32
ABO + PMT-301	164.9 ± 1.3	129.7 ± 4.7	4.84 ± 10^9^ ± 1.47 ± 10^8^	26.0	104.39
ABO + PMT-302	166.0 ± 2.5	134.5 ± 2.9	4.23 ± 10^9^ ± 1.74 ± 10^8^	28.4	98.64
ABO + PMT-303	155.6 ± 3.8	133.8 ± 5.5	2.56 ± 10^9^ ± 6.82 ± 10^7^	17.0	68.63

The NTA size distribution data is essentially spectral data whereby different peaks point out the predominant particle sizes in the measured solution. However, it is rather tedious to distinguish the prevalent sizes the different AβO + stilbene PAL combinations yield by only visually inspecting the size distribution charts. Therefore, we implemented a principal component analysis (PCA) in order to unveil whether there were certain trends in the observed particle sizes and if these trends potentially correlate with other measurements of PAL-induced structural adaptation. Briefly, PCA is a dimensionality reduction technique for multivariate data sets to simultaneously capture as much variability as possible and conserve the pertinent information responsible for the major sources of variability (Abdi and Williams, 2010). Analysis of the NTA results reveals that 86.5% of the total variance is captured using two principal components (PC1 and PC2). Figure 8B displays the 2-dimensional PC score plot that highlights the magnitude of variance and groupings within the NTA size distribution data. One single marker in the 2D score plot represents an average of 3 independent size distribution measurements by NTA. Thus, for instance the AβO only group comprises the data from 18 independent NTA measurements. Collectively both PC1 and PC2 loading spectra produce peaks that encapsulate the majority of variance within the NTA size distribution data.

Intriguingly, each stilbene PAL group is clearly distinguished from the AβO alone group (red markers), except the AβO + PMT-401 (green markers) group. This feature indicates that each stilbene PAL treatment influences the AβO size distribution to some extent, although PMT-401 treatment produces very similar size distribution profile with the AβO alone group (the red and green groups highly overlapping). This result aligns with our other measurements showing the comparably poor characteristics of PMT-401 in AβO modulation. Furthermore, AβO treated with PMT-402 (blue markers) is the most discernible group from the others, which is also consistent with our other analyses. The 301–303 candidates not only differentiate from untreated AβO, but also from each other, with a slight overlapping of the AβO + PMT-301 (black markers) and AβO + PMT-302 (magenta markers) groups. The AβO + PMT-303 group (cyan markers) is noticeably tight compared to the others reflecting a significantly low internal variation.

As the PC1 and PC2 loading spectra are investigated, PMT-301, PMT-302 and PMT-402 have mostly positive score values along the PC1 axis of the score plot, corresponding to their intensities at 115 and 145 nm. Therefore, it is evident that these PALs produce more AβOs of approximate diameter of 115 and 145 nm than Aβ-alone, PMT-401, and PMT-303. Moreover, the PMT-402 group has markedly positive scores along the PC2 axis, and the corresponding PC2 loading spectrum possesses a prominent positive peak at 105 nm. This is indicative of PMT-402 also generating a subpopulation of 105 nm particulates. On the other hand, the majority of PMT-301 and PMT-302 measurements have negative score values along the PC2 axis, and the PC2 loading spectrum displays two negative peaks at 125–135 and 185 nm region. Taken together, PMT-301 and PMT-302 yield more particles of these sizes more than the other stilbenes.

## 4 Discussion

In aqueous solution, Aβ forms a large size and conformational distribution that is highly influenced by its method of preparation (Teplow, 2006). Soluble Aβ can assume multiple states, with various oligomeric states implicated in different aspects of AD pathogenesis (Lesné et al., 2006; Amar et al., 2017; De et al., 2019). However, the correlation of a specific oligomeric size to a specific cellular target is difficult to establish, as very large assemblies can be overlooked in sieving methods and the dynamic nature of AβO makes its state highly sensitive to reagent and matrix influences (Hepler et al., 2006; Hubin et al., 2014). A general consensus across studies points to intermediate-sized (>50 kDa), A11-positive oligomers inducing the broadest range of neuronal toxicity and dysfunction (Vandersteen et al., 2012; Cline et al., 2018). We therefore employed a simple prep that produces A11-positive, neurotoxic oligomers, and can likewise reproducibly transition into a soluble protofibril species. These oligomers retain a significant amount of conformational heterogeneity and ∼40% beta sheet content (Sandberg et al., 2010; Vandersteen et al., 2012; Roy et al., 2017).

Our previous reports on a fluorene-based PAL compound demonstrated its high potency is related to the localization of nitroxide antioxidant activity within the cell, interrupting the cycle of ROS-enhanced AβO cytotoxicity and accumulation (Petrlova et al., 2012; Altman et al., 2015; Hilt et al., 2018). The high antioxidant potency of the nitroxide can be attributed its ability to cycle through alternate redox states, mimicking the antioxidant defense of superoxide dismutase (SOD) (Hideg and Kálai, 2007; Mandal et al., 2007). In cells, the redox cycle for a nitroxide begins with the reduction of the N-O state to the N-OH state, which can be re-oxidized to N-O by ROS. Likewise, hydroxyl and peroxyl radicals can oxidize the N-O state to the nitrone (N=O), which is then available to remove superoxide and regenerate the N-O state. Subsequent reduction (e.g., by GSH) to N-OH allows for a single nitroxide to perform several rounds of scavenging. Importantly the N-oxyl (nitroxyl) is not oxidative to other biomolecules (lipids, proteins, DNA). Consistent with previous studies of a fluorene-based PAL, the synergistic activity of stilbene PALs provides a potency 10–500 times greater than other anti-amyloid small molecules such as SEN1269 (Scopes et al., 2012) and resveratrol (Marambaud et al., 2005).

The antioxidant activity of the PALs is exemplified by the strong attenuation of the CellROX signal in MC65 cells induced for C99 expression. This potency is significant for two reasons: 1) brain bioavailability is a challenge for nearly all agents targeting amyloid-like proteins, and 2) it facilitates a targeted mechanism for countering oxidative stress, which is important as high antioxidant doses can become prooxidants, down-regulate endogenous antioxidant pathways and interfere with beneficial ROS/RNS-dependent signaling (Bouayed and Bohn, 2010).

Because each of the stilbene PAL agents contain a similar nitroxyl moiety, their effects on AβO conformation and assembly can point to structures that are superior in AβO engagement and/or remodeling. However, caveats remain, such as differences in the availability of free compound to the cell interior (e.g., differences in permeation, off-target protein binding). In terms of bioactivity and various metrics of AβO engagement, the aminostilbene PMT-402 displays the highest potency. In contrast, the aminostilbene PMT-401 shows the lowest potency. Although care was taken to maximize compound dispersion in all assays, the markedly lower solubility of PMT-401 could lower its effective concentration in our measurements.

Recognition of oligomer species by the A11 antibody provides a basis for identifying conformational toxicity, as it reacts against a diverse set of protein aggregates known to trigger neurodegeneration (Kayed et al., 2003). As described previously (Necula et al., 2007), attenuation of A11 binding can help identify compounds that convert AβO towards a less toxic conformation. Each of the 5 stilbene PALs significantly reduce A11 binding to AβO. The results suggest PMT-402 is most potent in this regard, however the variability inherent to the assay precludes ranking the other PAL agents according to potency. The reduction in binding may reflect an allosteric transition of the peptide away from its A11-positive conformation, although PAL occupation of the A11 epitope is also possible.

The CD spectrum of the aqueous AβO sample prior to its 1-h incubation displays a relatively flat, low amplitude signal (not shown) that increase in β-sheet content with time (Clements et al., 1996; Shea et al., 2019). This characteristic signal has been attributed to the peptide in the extended α-sheet, a conformation predicted to populate “toxic” soluble oligomers that ultimately mature into amyloid fibrils (Hayward and Milner-White, 2008; Babin et al., 2011; Hopping et al., 2014; Shea et al., 2019). After 1-h of incubation, the CD spectrum of the Aβ oligomers used in this study indicates the major fraction of secondary structure falls within the “disordered” portion of the spectra, however non-random conformations such as the extended poly-proline-II (PPII)-like helix also contribute intensities in this regime (i.e., a band with defined minimum in the 195–200 nm) (Gokce et al., 2005; Sandberg et al., 2010; Nisbet et al., 2013).

Deconvolution of the CD spectra for secondary structure composition indicates that PAL treatment of AβO with PALs in general results in a slight reduction in β-strand and corresponding increase in the helical component, with the major spectral change occurring within the regime classified as “other.” The most significant effect is, with the exception of PMT-401, is the generation of a polyproline II (PPII)-like spectral intensity (Adzhubei et al., 2013). A similar result has been observed when treating AβO with a fluorene-based PAL (Altman et al., 2015), or examining the peptide at low temperature (Danielsson et al., 2005). Both the α-sheet and PP-II configurations display exposed backbone carbonyls, which have been postulated to drive protein-protein interactions (Fernández and Crespo, 2008; Hayward and Milner-White, 2008; Theillet et al., 2013; Adzhubei et al., 2017), readily convert to β-strand (Blanch et al., 2000; Hayward and Milner-White, 2008; Adzhubei et al., 2017), and have been proposed to illicit “toxic” conformations (Blanch et al., 2000; Shea et al., 2019). With respect to these populations within AβO, the CD results are consistent with a decrease in the fraction of α-sheet structure for the bioactive PALs.

We also examined the effect of the stilbene PALs on the CD spectrum of protofibril oligomers formed after 24-h incubation. These species display significantly more order with a high fraction of β-sheet secondary structure (Kayed et al., 2009). A far less dramatic alteration of the CD spectrum is observed with PAL treatment of protofibrils, although each of the compounds increase β-sheet content and decrease disordered content. With protofibrils showing low cytotoxicity, the absence of their disruption by the stilbene PALs can be viewed as a favorable property.

With respect to dye binding, compared to ThT, changes in NR fluorescence intensity appear to provide a better predictive value for the potency in the MC65 assay. In contrast to the CD measurements PMT-402 is not distinguished in either dye assay. Each of the PAL agents provide a strong inhibition of b-sheet formation as determined by ThT. However, the NR assay reports a differential effect with PMT-302, with the compound producing increased NR solvent exposure. Thus, the ability of PALs to occlude hydrophobic patches within AβO may provide a useful metric in identifying candidates to protect against AβO toxicity.

As shown in previous studies (Petrlova et al., 2012; Altman et al., 2015), EPR analyses of both AβO^TOAC^ and protofibril AβO^TOAC^ demonstrate that the TOAC spin label at position 26 reflects a moderate degree of order within the oligomer. Although the TOAC label is diluted with the oligomer to minimize spin-spin interaction, dipolar interactions between labels in close (∼1.5 nm) proximity also contribute to the spectral broadening. Thus, we cannot attribute increased motional freedom as the only source behind the PAL-induced effects onto the AβO^TOAC^ and protofibrils (Petrlova et al., 2012; Altman et al., 2015). In any event, both an increase in the spin label correlation time and decreased dipolar coupling are indicative of oligomer remodeling. PMT-301 and PMT-303 induce the largest effects on the EPR spectrum of AβO^TOAC^, while PMT-402 had the greatest effect in the protofibril Aβ^TOAC^ sample. Interestingly, PMT-401 showed no ability to alter the structure around the TOAC spin label. Therefore, EPR spectral changes reported by the TOAC label at position 26 may also be predictive for active candidates in the MC65 assay. Furthermore, the subtraction of the free PAL component from the PMT-401 treated AβO^TOAC^ results in a negative spectral line. This can be explained if the PMT-401 contribution to the composite sample is broadened *via* dipolar coupling. Thus, PMT-401 may uniquely position its nitroxyl moiety in close proximity position 26 of the AβO^TOAC^ sample. Finally, the EPR results again identified PMT-302 and PMT-401 as the least consequential PALs, showing a general agreement among the bioactivity, CD and dye binding results.

While the explicit molecular level mechanisms on how the stilbene PALs give rise to specimens of different sizes remain to be solved, the NTA-PCA analysis reveals some noteworthy aspects. First, PMT-401 (least effective in bioactivity) most resembles the untreated AβO; and PMT-402 (most effective in bioactivity) is the most distinguished in the PCA plot. Also, the FWHM feature of the distribution curve is somewhat predictive (Table 2), with PMT-402 and PMT-303 showing the lowest values (the high FWHM value of PMT-301 – intermediate bioactivity–suggests this metric may not serve as predictive in all cases). Second, with the exception of PMT-401, all the stilbene PAL modulators yield unique size distribution profiles that are markedly different from the AβO-alone sample and across each other as well. Therefore, the neural cell protective nature of the potent stilbene PALs may at least partially be explained by their capability to either 1) dislodge very large AβOs and protofibrils (>200 nm) into smaller particulates (105–145 nm), 2) assemble smaller toxic AβO (oligomers of 50–200 kD) into larger entities (105–145 nm), or 3) stabilize the existing less-toxic AβOs in the approximate size range of 105–145 nm. We cautiously hypothesize that whichever is the mechanism, the ultimate benefit is to generate AβOs of suitable size that are more easily dispatched from the cells. However, these hypotheses warrant for further targeted studies that were out of the scope of this study.

The amyloid-independent anti-inflammatory activity on HBMECs challenged by TGRL lipolysis products (TL) provides insights as to whether the antioxidant activity of the stilbene PALs can reduce inflammation on a more general basis. Only PMT-401 significantly suppressed TL-induced expression of all measured cytokines as well as COX-2 gene expression. Because PMT-401 is significantly more hydrophobic than the other PALs, effective downregulation of inflammation in the HBMEC model requires is likely dependent on the agent’s partition into lipophilic domains. The other PALs did not suppress cytokine gene expression, however PMT-302 and PMT-303 did significantly suppress TL-induced COX-2 expression.

The fact that the secondary structure of the PAL-treated AβO remains largely disordered implies that shifting the equilibrium away from the toxic conformer may only require the modulation of a discrete structural feature within the ensemble. As the clinical quantification of AD risk *via* early biomarkers becomes a reality, small molecules are practical for continuous treatment to maintain a favorable balance of Aβ species (with respect to toxicity cellular clearance) in patients. This concept is similar to the administration of statins in cardiovascular health. In this regard, describing the interaction of our distinct PAL agents as a function of conformational toxicity will aid future development of small molecule structural correctors.

Developing small molecules with bifunctionality provides meaningful advantages as potential clinical applications are considered. Because *in vivo* safety and efficacy testing (e.g., tolerance, pharmacokinetics, pharmacodynamics) are carried out for single agents, there is a growing interest in developing compounds to address more than one target (Corson et al., 2008; Bachurin et al., 2017; Ramsay et al., 2018). The bi-functional approach provides synergistic action on two pathogenetic hallmarks of the disease resulting in considerable enhancement of the overall pharmacological effect and may provide both cognition-stimulating and disease-modifying actions.

## Data Availability

The original contributions presented in the study are included in the article/Supplementary Material, further inquiries can be directed to the corresponding author.
