# Supplementary material for: Novel Stilbene-Nitroxyl Hybrid Compounds Display Discrete Modulation of Amyloid Beta Toxicity and Structure
Source: Front Chem. 2022 May 26;10:896386. doi: 10.3389/fchem.2022.896386 (PMC9204515; doi:10.3389/fchem.2022.896386)
Supplement: Supplementary file 1 [file DataSheet1.pdf]

Table S1. Sequences of the specific human primers utilized in the qRT-PCR measurements for inflammatory markers.

Gene	Primer sequence (5'-3')
GAPDH	Sense-CACCAACTGCTTAG
	Antisense-TGGTCATGAGTCCT
ATF3	Sense-TTCTCCCAGCGTTAACACAAAA
	Antisense-AGAGGACCTGCCATCATGCT
SELE	Sense-TGGCAATGAAAAATTCTCAGTCA
	Antisense-TCAAGGCTAGAGCAGCTTTGG
IL-6	Sense-CACCGGGAACGAAAGAGAAG
	Antisense-TCATAGCTGGGCTCCTGGAG
IL-8	Sense-CCTTTCCACCCCAAATTTATCA
	Antisense-TGGTCCACTCTCAATCACTCTCAG
COX-2/PTGS2	Sense-CTGAATGTGCCATAAGACTGACCT
	Antisense-TCCACAGATCCCTCAAAACATTT

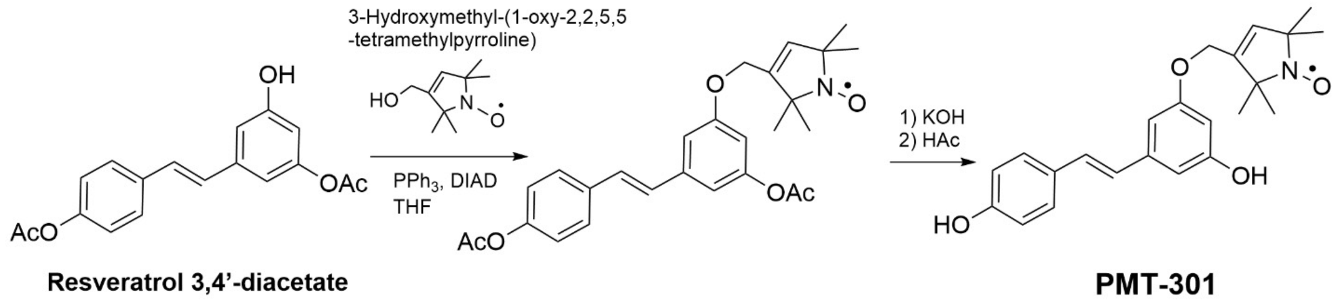

Figure S1. Synthesis of PMT-301

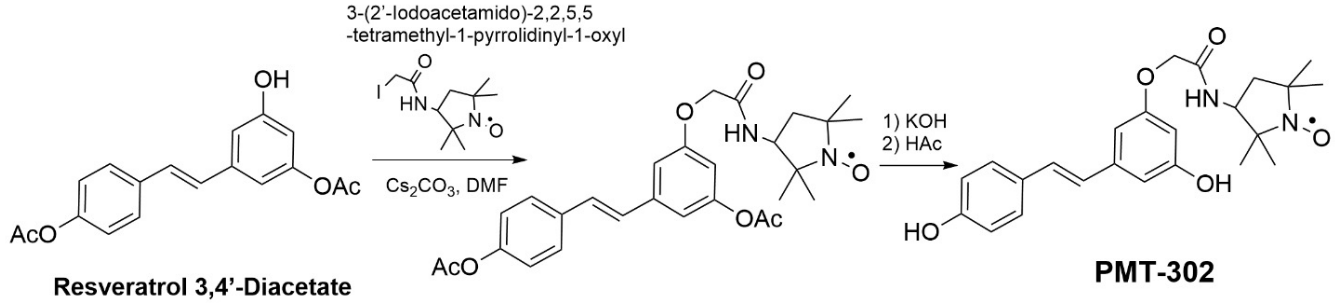

Figure S2. Synthesis of PMT-302

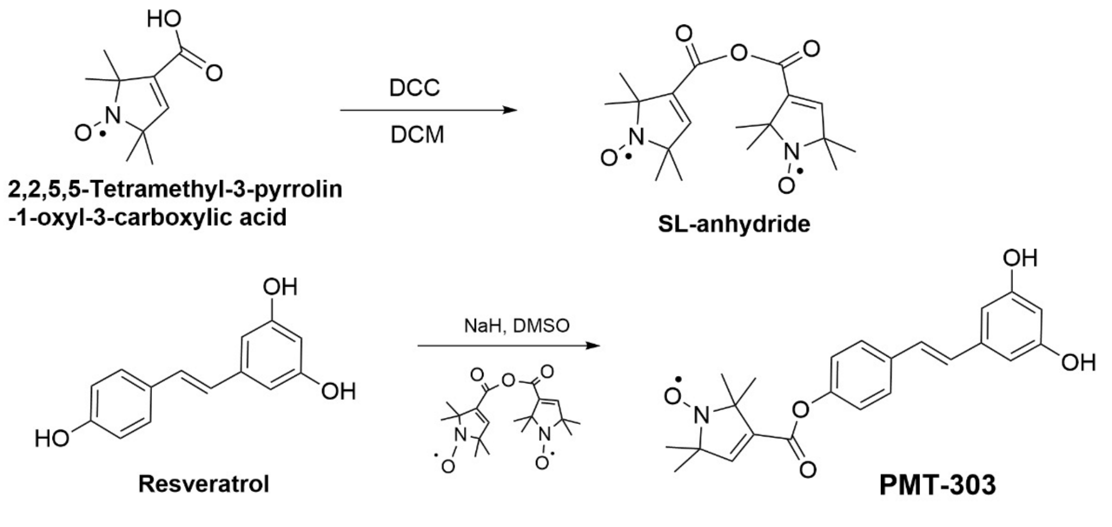

Figure S3. Synthesis of PMT-303

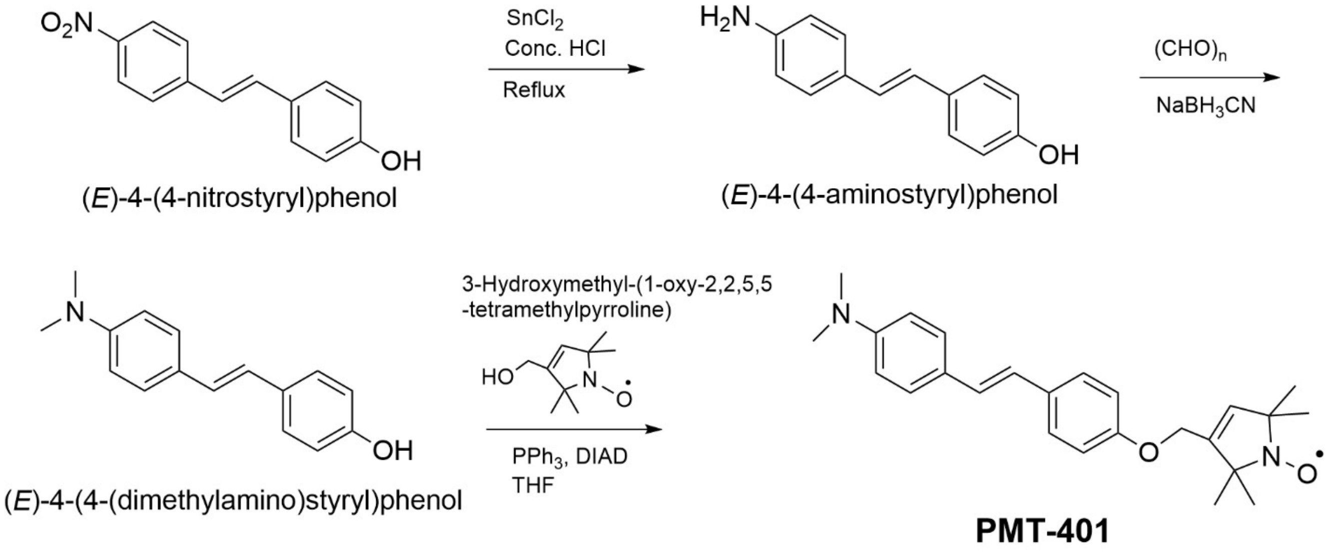

Figure S4. Synthesis of PMT-401

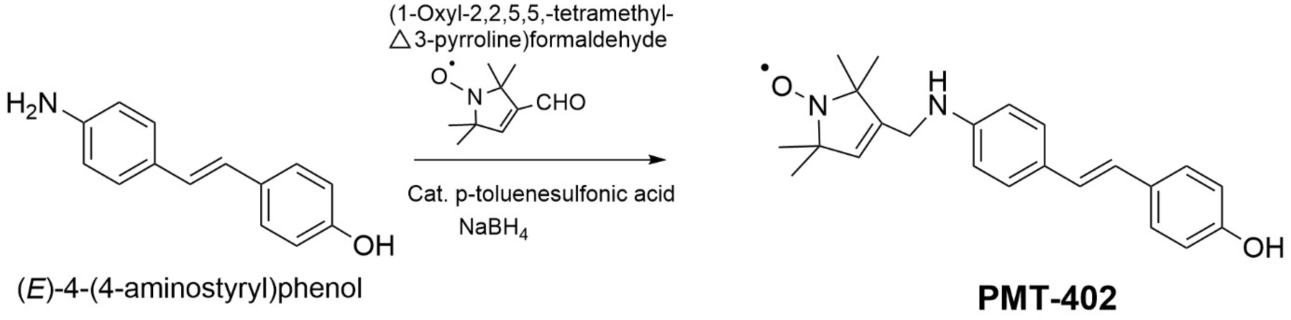

Figure S5. Synthesis of PMT-402

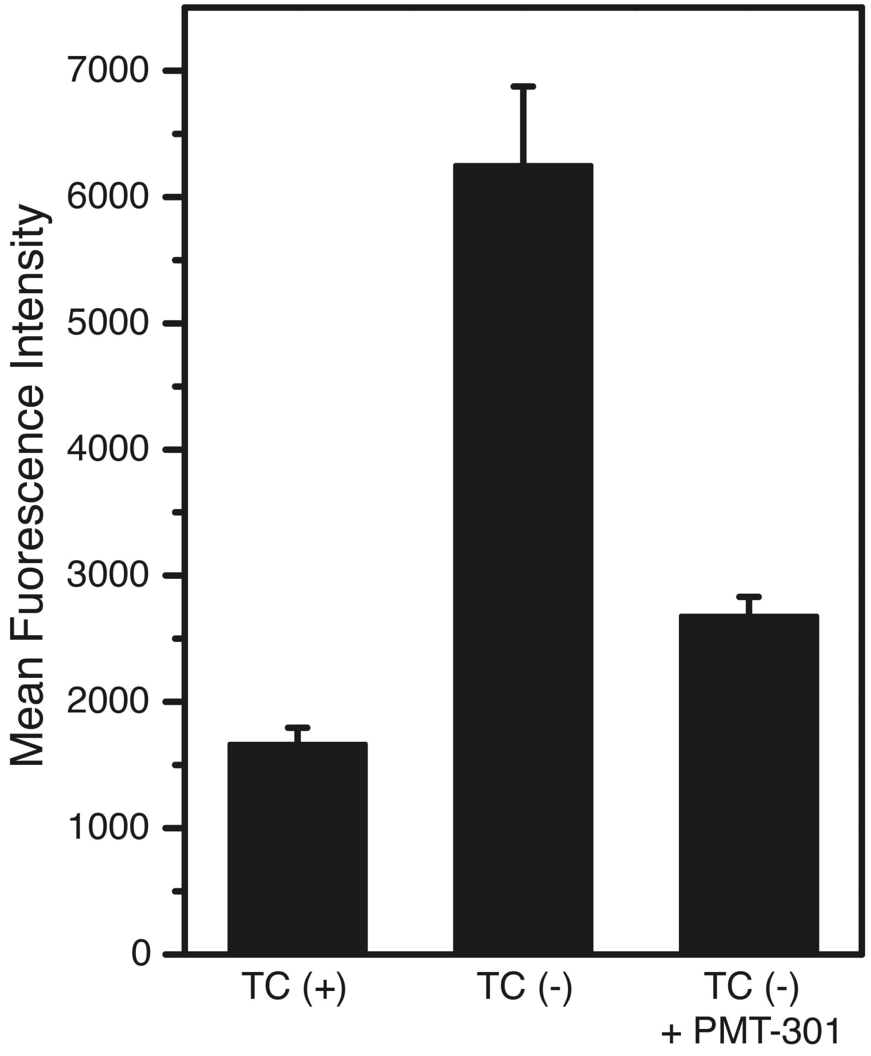

Figure S6. Intensity comparison of CellROX emission in MC65 cells. Triplicate measurements of the mean mean fluorescence intensity were done in 3 randomly selected areas of each of the cell culture fields, with background correction.

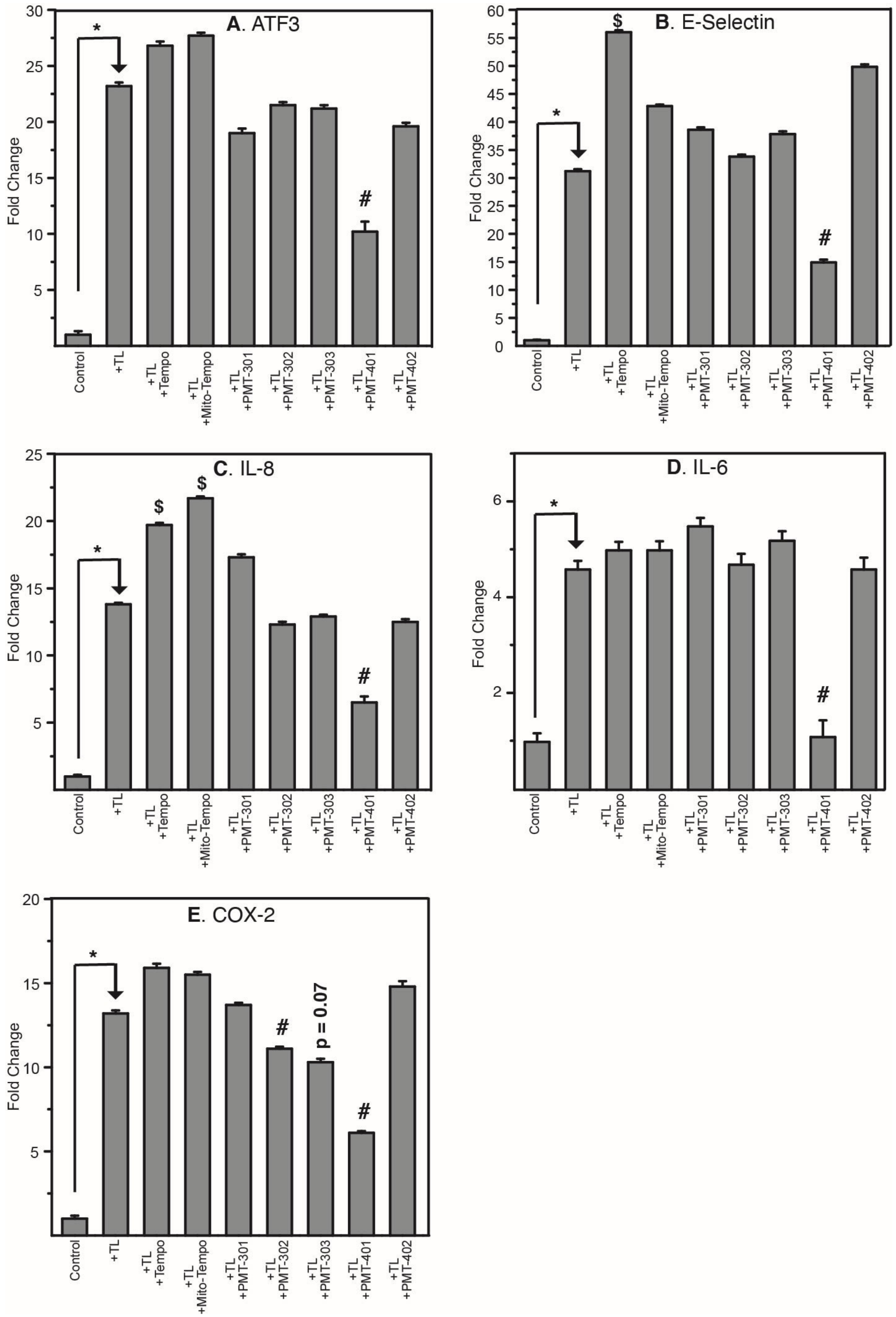

Figure S7. qRT-PCR measurements for inflammatory markers in HBMEC cells with and without PAL treatment. In this model, TGRL lipolysis products (TL) are used to upregulate stress-responsive transcription factors ATF3, COX-2, and proinflammatory genes (IL-6, IL-8 and E-selectin).
